# Perineural invasion in pancreatic cancer: proteomic analysis and *in vitro* modelling

**DOI:** 10.1002/1878-0261.12463

**Published:** 2019-03-05

**Authors:** Wasfi Alrawashdeh, Richard Jones, Laurent Dumartin, Tomasz P. Radon, Pedro R. Cutillas, Roger M. Feakins, Branko Dmitrovic, Ihsan Ekin Demir, Guralp O. Ceyhan, Tatjana Crnogorac‐Jurcevic

**Affiliations:** ^1^ Centre for Molecular Oncology Barts Cancer Institute Queen Mary University of London UK; ^2^ MS Bioworks, LLC Ann Arbor MI USA; ^3^ Centre for Haemato‐Oncology Bart Cancer Institute Queen Mary University of London UK; ^4^ Department of Histopathology Royal London Hospital UK; ^5^ Department of Pathology and Forensic Medicine Faculty of Medicine University of Osijek Croatia; ^6^ Department of Surgery Klinikum rechts der Isar Technische Universität Munich Germany

**Keywords:** microdissection, pancreatic cancer, perineural invasion, proteomics, VGF

## Abstract

Perineural invasion (PNI) is a common and characteristic feature of pancreatic ductal adenocarcinoma (PDAC) that is associated with poor prognosis, tumor recurrence, and generation of pain. However, the molecular alterations in cancer cells and nerves within PNI have not previously been comprehensively analyzed. Here, we describe our proteomic analysis of the molecular changes underlying neuro‐epithelial interactions in PNI using liquid chromatography–mass spectrometry (LC‐MS/MS) in microdissected PNI and non‐PNI cancer, as well as in invaded and noninvaded nerves from formalin‐fixed, paraffin‐embedded PDAC tissues. In addition, an *in vitro* model of PNI was developed using a co‐culture system comprising PDAC cell lines and PC12 cells as the neuronal element. The overall proteomic profiles of PNI and non‐PNI cancer appeared largely similar. In contrast, upon invasion by cancer cells, nerves demonstrated widespread plasticity with a pattern consistent with neuronal injury. The up‐regulation of SCG2 (secretogranin II) and neurosecretory protein VGF (nonacronymic) in invaded nerves in PDAC tissues was further validated using immunohistochemistry. The tested PDAC cell lines were found to be able to induce neuronal plasticity in PC12 cells in our *in vitro* established co‐culture model. Changes in expression levels of VGF, as well as of two additional proteins previously reported to be overexpressed in PNI, Nestin and Neuromodulin (GAP43), closely recapitulated our proteomic findings in PDAC tissues. Furthermore, induction of VGF, while not necessary for PC12 survival, mediated neurite extension induced by PDAC cell lines. In summary, here we report the proteomic alterations underlying PNI in PDAC and confirm that PDAC cells are able to induce neuronal plasticity. In addition, we describe a novel, simple, and easily adaptable co‐culture model for *in vitro* study of neuro‐epithelial interactions.

**Abbreviations**
GAP43: Neuromodulin
IPA: ingenuity pathway analysis
LAMB1: Laminin B1
LAMC1: Laminin C1
LC‐MS/MS: liquid chromatography–mass spectrometry
MAP1B: microtubule‐associated protein 1B
NES: Nestin
NGF: nerve growth factor
PDAC: pancreatic ductal adenocarcinoma
PNI: perineural invasion
SCG2: secretogranin II
TNC: Tenascin

## 1 Background

Perineural invasion (PNI) is a characteristic feature of pancreatic adenocarcinoma (PDAC), which is, upon detailed histopathology examination, encountered in almost 100% of cases (Liu, 2002; Marchesi *et al*., 2010; Pour *et al*., 2003). PNI correlates with tumor recurrence (Chou *et al*., 2013; Kayahara *et al*., 1993; Kelsen *et al*., 1997; Liu, 2002; Shimada *et al*., 2011), is implicated in generation of pain (Bapat *et al*., 2011; D'Haese *et al*., 2014), and confers poor prognosis (Zhang *et al*., 2013). However, despite the first description of PNI more than 150 years ago (Demir *et al*., 2012a) and its doubtless importance, it is still the least studied route of metastatic spread, and our knowledge of the neuro‐epithelial interactions in PDAC remains limited and fragmented. Recent reports indicated that the microenvironment created by this interaction provides a suitable milieu for tumor growth, resulting in enhanced proliferation and inhibition of apoptosis as well as increased migration of cancer cells (Bapat *et al*., 2011; Dai *et al*., 2007). At the same time, akin to neoangiogenesis and lymphangiogenesis, tumor cells also promote neoneurogenesis, manifested in increased nerve hypertrophy and neural density in human PDAC tissues (Ceyhan *et al*., 2009a). The presence and implication of alterations in the neuronal structure and/or function, that is, neuronal remodeling and plasticity, in the context of cancer are increasingly recognized (Demir *et al*., 2015). Moreover, neuroplastic changes are seen in early development of PDAC with ablation of sensory neurons leading to slower development of PanINs (pancreatic intraepithelial neoplasia) (Saloman *et al*., 2016), while in contrast, subdiaphragmatic vagotomy in LSL‐Kras+/G12D; Pdx1‐Cre (KC) mice accelerated PDAC development (Renz *et al*., 2018a). Furthermore, antibody targeting of NGF (nerve growth factor) or small molecule blocking of NGF‐Trk pathway led to inhibition of cancer progression and increased overall mice survival in LSL‐Kras+/G12D; LSL‐Trp53+/R172H; Pdx1‐Cre (KPC) mouse model (Renz *et al*., 2018b; Saloman *et al*., 2018). These findings confirm the important and active role of the nervous system in both PDAC development and progression as well as its potential for therapeutic targeting (Demir *et al*., 2012b; Jobling *et al*., 2015; Mancino *et al*., 2011; Pour *et al*., 2003; Saloman *et al*., 2016; Stopczynski *et al*., 2014).

Most of the observations on PNI were made using a currently limited number of *in vitro* and *in vivo* models (Amit *et al*., 2016; Deborde *et al*., 2018; Demir *et al*., 2012a; Liebig *et al*., 2009), and very few large‐scale studies of PNI tissues have been performed until now: Gene expression of microdissected PNI and non‐PNI salivary adenoid cystic carcinoma cells (Chen *et al*., 2007), and gene and microRNA profiles were analyzed in bulk prostate cancer tissues from cases with or without PNI (Prueitt *et al*., 2008). In the pancreas, transcriptome and proteome of high and low nerve‐invasive PDAC cell lines injected in NOD/SCID (nonobese diabetes/severe combined immunodeficient) mice were described (Abiatari *et al*., 2009; Hibi *et al*., 2009; Koide *et al*., 2006), but no large‐scale molecular data from cancer cells or nerves within PNI tissues are available as yet. Here, we report on global proteomic analysis of laser microdissected, matched PNI and non‐PNI cancer as well as invaded and noninvaded nerves from human formalin‐fixed, paraffin‐embedded (FFPE) tissues. In addition, we developed an *in vitro* model that successfully recapitulated several phenotypic and molecular alterations observed in PNI in human PDAC tissues.

## 2 Materials and methods

### 2.1 Tissues and cell lines

Normal and PDAC tissues were collected and obtained from the Department of Histopathology, Royal London Hospital (London, UK), Department of Pathology and Forensic Medicine (Osijek, Croatia) and from European Pancreas Centre Research Laboratory at the University of Heidelberg (Heidelberg, Germany). All tissues were obtained with full ethical approval from the respective Institutional Boards and used in accordance with the Human Tissue Act 2004.

PDAC cell lines (MiaPaca2, BxPC3, and Capan1) were obtained from Cancer Research UK Tissue Culture Service and routinely cultured in Dulbecco's modified Eagle's medium, DMEM (Invitrogen, Paisley, UK) supplemented with 10% heat‐inactivated fetal bovine serum (FBS) (Autogen Bioclear, Wiltshire, UK) and penicillin/streptomycin at 37 °C in 5% CO_2_. PC12 cells, a rat pheochromocytoma cell line, were a kind gift from Dr Lesley Robson (Blizard Institute, QMUL London) and were grown in poly‐d‐lysine hydrobromide (PDL; MW 30 000–70 000, Sigma, Dorset, UK P7886) coated tissue culture flasks in RPMI media supplemented with 10% heat‐inactivated horse serum, 5% heat‐inactivated FBS, and penicillin/streptomycin at 37 °C in 5% CO_2_. The identity of the cell lines utilized was confirmed by STR profiling.

### 2.2 Tissue section preparation and laser microdissection

Ten‐micrometer sections were cut from each of five FFPE PDAC blocks (PDAC 1–5, Table S1) and mounted on PEN membrane slides (Zeiss, Munich, Germany) (Fig. S1). Every fifth section was cut at 5 μm, routinely stained with H&E, and reviewed to determine areas for laser microdissection. After baking in an oven at 55 °C for 45 min, sections on PEN slides were deparaffinized using xylene and rehydrated in graded ethanol, stained with hematoxylin, and dehydrated through graded ethanol followed by xylene and air‐drying for 5 min. The staining procedure was completed in 15 min. Laser microdissection was performed using PALM instrument (Zeiss). PNI cancer, non‐PNI cancer, invaded nerves, and noninvaded nerves were separately dissected from each of the five pancreatic cancer cases and stored at −20 °C.

### 2.3 Protein extraction for proteomic analysis

Laser microdissected samples (approximately 30 000 cells) were resuspended in 20 μL liquid tissue buffer (Expression Pathology, Inc., Rockville, MD, USA), and protein extraction and digestion were performed according to the manufacturer's instructions. Briefly, samples were boiled for 90 min at 95 °C, cooled on ice, and digested overnight at 37 °C with sequencing grade trypsin (Promega, Southampton, UK) (1 μg/20 μL buffer). Peptide concentration was measured with Micro BCA kit (Thermo Fisher Scientific, Waltham, MA, USA), and DTT (10 mm final concentration) was then added and samples were boiled at 95 °C for 5 min before storing at −20 °C until further analysis.

### 2.4 Proteomic analysis and data processing

LC‐MS/MS was performed (MSBioworks, Ann Arbor, MI, USA) on a Nanoacquity LC system coupled to LTQ Orbitrap Velos platform (Thermo Fisher) using 1 μg peptide digests of each of the five matched PNI and non‐PNI cancers and invaded and noninvaded nerves, in duplicate. Buffer A was 0.1% FA in water, and buffer B was 0.1% FA in acetonitrile. Samples which were suspended in 0.1% FA were first loaded on an IntegraFrit trap column (75 μm × 30 mm) packed with Jupiter Proteo C12 material with 5 μm particle size (Thermo Fisher Scientific). Peptides were then eluted from a 75 μm × 250 mm, 4 μm particle Jupiter Proteo C12 analytical column (Phenomenex, Torrance, CA, USA) at 350 nL·min^−1^ using 5–22% B over 70 min then to 35% B over 20 min and to 85% B over 15 min. Total run time was 2 h/sample. Data were acquired over mass range 300–1600 in a data‐dependent manner. Each full MS scan was followed by MS/MS of the 15 most abundant ions. Dynamic exclusion and repeat settings ensured each ion was selected only once and excluded for 30s thereafter.

MS data were analyzed using maxquant version 1.4.0.1 (Cox and Mann, 2008). The proteins were identified by searching MS and MS/MS data of peptides against the SWISS‐PROT human canonical database (20 556 entries) appended with common contaminants using the Andromeda search engine. Oxidation of methionine and protein N‐terminal acetylation was chosen as variable modifications. The minimum peptide length was specified to be seven amino acids. The initial maximal mass tolerance in MS mode was set to 7 ppm, whereas fragment mass tolerance was set to 0.5 Da for fragmentation data. The maximum false protein and peptide discovery rate (FDR) were specified as 0.01 and determined using a decoy database. Proteins were identified with ≥2 peptides/protein.

### 2.5 Estimation of laser microdissected sample purity

We attempted to estimate the degree of contamination based on the assumption that for a neuronal protein, for example, the level of expression in the PNI cancer relative to the invaded nerves is indicative of the degree of contamination of cancer sample by the adjacent nerve. We identified four known neuronal markers in the nerves dataset: glial fibrillary acidic protein (GFAP), S100B, neurofilament heavy polypeptide (NEFH), and neurosecretory protein VGF. We first calculated the abundance of each protein relative to the sample in which it was identified (for both PNI cancer samples and invaded nerves samples). The obtained value for each protein in the PNI cancer sample was then expressed as a percentage of its abundance in the matching invaded nerve sample. While most of these proteins were not detected in most PNI cancer samples, the relative abundance varied between the four proteins in the same sample pair (PNI cancer and invaded nerve) as well as for the same protein in different sample pairs particularly for GFAP.

We then used the relative abundance of the four proteins in each sample pair to estimate the degree of contamination of the PNI cancer sample by the matching invaded nerves’ sample. We were not able to identify any epithelial‐specific protein in the invaded nerves samples to attempt a similar approach to estimate purity of invaded nerves samples.

### 2.6 Immunohistochemistry

Basic clinico‐pathological data of FFPE tissue samples used for Immunohistochemistry (IHC) analysis are summarized in Table S1. Four‐micrometer tissue sections were stained using rabbit anti‐SCG2 primary antibody (dilution 1 : 500 HPA011893 Prestige Antibodies, Sigma‐Aldrich) and anti‐VGF primary antibody (1/400 dilution, Abcam ab69989; Milton, Cambridge, UK). SCG2 IHC was performed in Ventana Discovery automated system, while VGF was performed manually with Vectastain ABC kit (Vector Labs, Peterborough, UK) according to the manufacturer's instructions. Staining intensity was semiquantitatively assessed at 200× magnification as absent (0), weak (1), moderate (2), or strong (3).

### 2.7 Western blotting

Cell lysis was performed using NP‐40 buffer (1% NP‐40, 50 mm Tris pH 7.4, 150 mm NaCl) with protease inhibitors (Roche Diagnostics, West Sussex, UK). Twenty‐five micrograms of protein lysate was analyzed by SDS/PAGE as previously described (Whiteman *et al*., 2007).

Following antibodies were used: goat anti‐VGF (Santa Cruz, R‐15:sc‐10383; Germany) 1 : 800; rabbit anti‐GAP43 (Abcam ab16053, Milton) 1/20 000; mouse anti‐GAPDH (Santa Cruz, Germany) 1/20 000; mouse anti‐Nestin, (Abcam, Milton), 1 : 200; and rabbit anti‐Caspase 3 ( Cell Signaling, 9662S; Leiden, the Netherlands) 1 : 400). Band intensities were quantified in imagej (National Health Institute, https://imagej.nih.gov/ij/) relative to the intensity of the GAPDH in the same sample. Densitometry results of three independent experiments were expressed as a fold change relative to the control samples.

### 2.8 PC12 co‐culture experiments

For the Transwell co‐cultures, 1.5 × 10^5^ PC12 cells were seeded in complete RPMI media in 6‐well plate precoated with PDL. 1.5 × 10^5^ PDAC or PC12 cells were also seeded in their respective media in 6‐well plate inserts (1 μm pore size, BD Biosciences, San Jose, CA, USA) in separate plates. On the next day, media was removed and cells in inserts and wells were washed three times with serum‐free RPMI. Inserts with PC12 or PDAC cells were then combined with wells containing PC12 cells before adding serum‐free RPMI to wells (2 mL) and inserts (1 mL). Twenty four hours later, phase‐contrast photographs of PC12 cells in the wells were taken using an inverted Olympus CKX41 microscope (Olympus, Hamburg, Germany) at 200× magnification. PC12 cells were then harvested for RNA and protein extraction and flow cytometry.

For the contact co‐cultures, 1.2 × 10^6^ PC12 cells were seeded in their complete RPMI media in T75 flasks precoated with PDL. On the next day, media was removed and cells were incubated with fresh media containing 2.5 μm CellTracker Green CMFDA (Invitrogen, Loughborough, UK) at 37° for 30 min. Cells were then washed twice with serum‐free RPMI media and 1.2 × 10^6^ PDAC, or PC12 cells in complete RPMI media were added to the flasks. The added cells were then allowed to attach for 2 h. Co‐cultures were washed three times before adding 24 mL of fresh serum‐free RPMI. After 22 h, cells were harvested, and CMFDA‐positive PC12 cells were separated using FACS. The positively sorted cells were checked again using FACS to verify purity, and an aliquot of those cells was seeded on coverslips for additional immunocytochemical validation of their purity.

Seeding density (~15 000 cells/1 cm^2^), ratio of PDAC to PC12 cells (1 : 1), and ratio of media to cells (1 mL/1.0 × 10^5^) were kept constant, and both co‐cultures were performed and analyzed in parallel

### 2.9 Preparation of conditioned media

Conditioned media were prepared from 4 × 10^6^ PDAC or PC12 cells after an overnight culture. Cells were washed three times before adding fresh serum‐free RPMI for conditioning. Twenty‐four hours later, media were collected, centrifuged at 4000 ***g*** for 5 min, filtered (0.22‐μm filters, Millipore, Cork, Ireland), and immediately used to treat PC12 cells.

### 2.10 Quantitation of PC12 neurite growth

Randomly selected high‐power fields (three per well, at 200× magnification) of PC12 were analyzed for neurite growth in imagej. The degree of neurite extension was estimated by counting the number of cells bearing neurites longer than the maximum diameter of the cell body (Katoh *et al*., 2000). At least 200 cells were counted in each well, and results were expressed as percentage of total number of cells. Each experiment was performed at least three times.

### 2.11 Annexin V/DAPI flow cytometry assay

Floating and attached cells were harvested, centrifuged, and washed with ice‐cold PBS, pelleted and resuspended in 100 μL Annexin binding buffer (10 mm HEPES, 140 mm NaCl, 2.5 mm CaCl_2_, pH 7.4) containing 5 μL of Annexin V Alexa Fluor 647 (Invitrogen). Following 15‐min incubation at room temperature in the dark, 400 μL of the binding buffer containing 1 μg·mL^−1^ 4′,6‐diamidino‐2‐phenylindole (DAPI) was added to each sample. Flow cytometry was immediately performed using BD Fortessa cell analyzer (BD Biosciences). PC12 cells growing in their complete media or serum‐free RPMI were included as negative and positive controls, respectively. Cells negative for both Annexin V and DAPI were quantified as the surviving fraction, and results were expressed relative to PC12 survival in complete media. Duplicate wells were analyzed within each experiment, and experiments were repeated three times.

### 2.12 RNA extraction, cDNA synthesis, and qRT–PCR

Total RNA was extracted using RNAqueous kit (Ambion, CA, USA). 1 μg of total RNA was reverse‐transcribed using Quantitech reverse transcription kit (Qiagen, West Sussex, UK). qRT–PCR was performed using SYBR green method on an ABI7500 cycler (Applied Biosystems, CA, USA). All primers were obtained from PrimerDesign (Southampton, UK). The VGF primer sequences were as follows: forward 5′‐TGAGACTTTGACACCCTTATCC and reverse 5′‐GGAACCGCCCAGGAATGA. Run conditions were as follows: 50 °C for 2 min, 95 °C for 15 min followed by 40 cycles of 95 °C for 15 sec, 60 °C for 30 sec, and 72 °C for 1 min. Data are presented as fold change relative to control after normalization to the mean of housekeeping genes B2M and RPL13 (PrimerDesign proprietary primer sequences).

### 2.13 VGF silencing

SiRNA knockdown of VGF in PC12 cells was performed using Lipofectamine RNAiMAX (Invitrogen) according to the manufacturer's instructions. SiRNA targeting VGF (On‐Target plus pool) or nontargeting siRNA (Dharmacon, Fisher Scientific, UK) was used at 1 nM final concentration. This was optimized to block the induced VGF expression but to maintain the basal levels of VGF expression at 24 h. Media was removed 6 h later, and cells were washed three times before incubation with PDAC‐ or PC12‐conditioned media for 24 h.

### 2.14 Statistical analysis

Differential expression of proteins was assessed with Student's t‐test using peak intensity values for label‐free relative quantitative analysis of the LC‐MS/MS data. For continuous data, Student's *t*‐test was used to compare means of data whereas ANOVA with post hoc correction for multiple comparisons was used when three or more samples were compared. For semiquantitative IHC data, Mann–Whitney test was used to compare the means, whereas Kruskal–Wallis test with post hoc correction for multiple comparisons was used when comparing more than two groups. Two‐tailed *P* values were always calculated, and results were plotted as mean ± standard error of mean (SEM). The statistical analysis was performed using graphpad prism 5.03 software (San Diego, CA, USA). The significance level was set at *P* < 0.05.

## 3 Results

### 3.1 Proteomic analysis of cancer cells and nerves in PNI

In order to assess the molecular alterations in cancer cells within the perineural environment, LC‐MS/MS analysis was performed on five matched PNI (cancer invading nerves) and non‐PNI (primary) cancer samples (Table S1). The estimated purity of the microdissected PNI cancer samples based on the average relative abundance of four neuronal marker proteins was 98 and 96% for PNI‐1 and PNI2, respectively, and ≥99% for the last three samples. We could not identify any epithelial‐specific markers in the nerve samples to assess the degree of their contamination, which would suggest even higher degree of purity of those samples.

**Figure 1:**
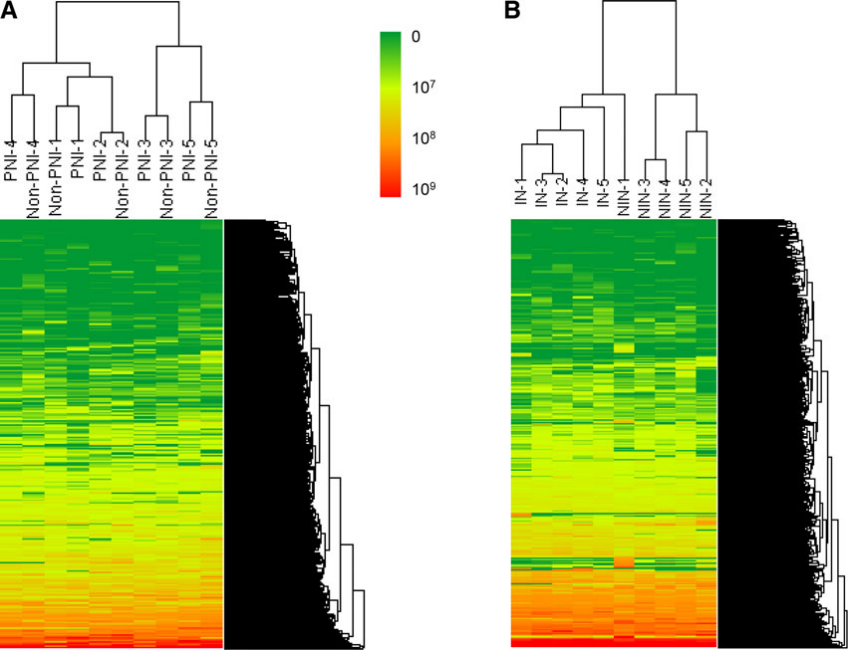
Hierarchal clustering of PNI and non‐PNI cancer samples (A) and invaded (IN) and noninvaded (NIN) nerve samples (B) according to their protein expression profiles. Each PNI sample clustered with its matching non‐PNI sample, while all invaded nerves clustered together. Only one noninvaded nerve grouped with invaded nerves. Clustering was performed using arraytrack™ software (FDA, Silver Spring, MD, USA). Scale represents peak intensities.

A total of 1628 nonredundant proteins were identified (Table S2), of which 1432 proteins were seen in perineural cancer cells from the PNI samples (1083–1266 per sample) and 1542 proteins in cancer cells from the non‐PNI samples (1092–1258 per sample). Only 31 proteins (1.9%) were differentially expressed (*P* ≤ 0.05) (Table S3); when a fold change of ≥1.5 was applied in addition to the *P* value, only eight up‐regulated and 11 down‐regulated proteins were identified. Using unsupervised hierarchical cluster analysis based on all identified proteins, PNI and non‐PNI samples obtained from each case were shown to cluster together, confirming further the similar nature of these samples (Fig. 1A). In parallel, LC‐MS/MS analysis of invaded and noninvaded nerve samples from the same five PDAC cases resulted in identification of 923 unique proteins (Table S4). In the invaded nerves, 847 proteins (681–729 proteins per sample) and in the noninvaded nerves 833 proteins (578–729 proteins per sample) were identified; 167 proteins (18%) were found differentially expressed (*P* ≤ 0.05) (Table S5). A list of 61 differentially expressed proteins selected for both *P* ≤ 0.05 and fold change of ≥2 is provided in Table 1. Interestingly, unsupervised hierarchal clustering revealed that all invaded nerves (IN1‐5) were grouped together and were separated from the four out of five noninvaded nerves (NIN2‐5) (Fig. 1B). This clustering pattern strongly suggested common underlying molecular changes upon neural invasion. While the reason for clustering of one of the noninvaded nerve samples, NIN‐1, with invaded nerves could potentially be due to invasion of those nerves at a different depth in the tissue sample (or even in the adjacent tissue block), the observed clustering pattern strongly suggested common underlying molecular changes upon neural invasion.

**Table 1:** List of proteins differentially regulated in invaded nerves compared to noninvaded nerves (two‐tailed Student's *t*‐test, *P* < 0.05 and FC > 2.0). The mean of peak intensity values from five samples is shown. IN: invaded nerves, NIN: noninvaded nerves, and FC, fold change

Protein ID	Gene name	Protein name	IN	NIN	*P* value	FC
P51659	HSD17B4	Peroxisomal multifunctional enzyme type 2	2.0E+06	0.0E+00	0	
O00231	PSMD11	26S proteasome non‐ATPase regulatory subunit 11	4.0E+06	0.0E+00	0.0001	
P50440	GATM	Glycine amidinotransferase, mitochondrial	2.0E+06	0.0E+00	0.0005	
P02647	APOA1	Apolipoprotein A‐I	2.0E+08	6.0E+08	0.001	0.4
P04040	CAT	Catalase	3.0E+07	6.0E+07	0.0012	0.4
P59768	GNG2	Guanine nucleotide‐binding protein G subunit gamma‐2	2.0E+07	7.0E+06	0.0017	2.3
P11166	SLC2A1	Solute carrier family 2	7.0E+06	5.0E+07	0.0036	0.2
P07339	CTSD	Cathepsin D	6.0E+07	3.0E+07	0.0037	2.0
Q8TAT6	NPLOC4	Nuclear protein localization protein 4 homolog	2.0E+07	2.0E+06	0.004	8.1
P54577	YARS	Tyrosine–tRNA ligase, cytoplasmic	2.0E+06	3.0E+05	0.004	7.6
P48681	NES	Nestin	4.0E+08	1.0E+08	0.0048	3.4
**P13521**	**SCG2**	**Secretogranin** II	**3.0E+07**	**3.0E+06**	**0.0064**	**9.6**
P19971	TYMP	Thymidine phosphorylase	6.0E+07	2.0E+07	0.0064	2.4
P08294	SOD3	Extracellular superoxide dismutase [Cu‐Zn]	3.0E+06	1.0E+07	0.0069	0.2
Q00325	SLC25A3	Phosphate carrier protein, mitochondrial	8.0E+06	2.0E+06	0.0072	3.4
P68871	HBB	Hemoglobin subunit beta; LVV‐hemorphin‐7	1.0E+09	8.0E+09	0.0078	0.2
P01857	IGHG1	Ig gamma‐1 chain C region	1.0E+08	3.0E+08	0.0079	0.4
P21796	VDAC1	Voltage‐dependent anion‐selective channel protein 1	1.0E+07	5.0E+06	0.008	2.4
P02730	SLC4A1	Band 3 anion transport protein	6.0E+05	1.0E+07	0.0083	0.0
**O15240**	**VGF**	**Neurosecretory protein VGF**	**1.0E+08**	**3.0E+07**	**0.0086**	**5.2**
P24821	TNC	Tenascin	5.0E+07	2.0E+07	0.0088	2.6
P11277	SPTB	Spectrin beta chain, erythrocyte	2.0E+05	2.0E+06	0.0098	0.1
Q9HCJ6	VAT1L	Synaptic vesicle membrane protein VAT‐1 homolog‐like	2.0E+07	5.0E+06	0.0111	2.8
P45880	VDAC2	Voltage‐dependent anion‐selective channel protein 2	1.0E+07	7.0E+06	0.0118	2.0
Q9UHG2	PCSK1N	ProSAAS; KEP	3.0E+07	6.0E+06	0.0123	4.7
P54920	NAPA	Alpha‐soluble NSF attachment protein	5.0E+06	2.0E+06	0.0123	2.2
Q01484	ANK2	Ankyrin‐2	5.0E+07	2.0E+07	0.0126	2.1
P36543	ATP6V1E1	V‐type proton ATPase subunit E 1	9.0E+06	4.0E+06	0.0133	2.3
O15075	DCLK1	Serine/threonine protein kinase DCLK1	4.0E+06	9.0E+05	0.0142	4.3
P06727	APOA4	Apolipoprotein A‐IV	3.0E+07	7.0E+07	0.015	0.5
Q9UI12	ATP6V1H	V‐type proton ATPase subunit H	6.0E+06	3.0E+06	0.0154	2.2
P02042	HBD	Hemoglobin subunit delta	1.0E+07	1.0E+08	0.0155	0.1
P00918	CA2	Carbonic anhydrase 2	5.0E+05	7.0E+06	0.0155	0.1
Q9NTK5	OLA1	Obg‐like ATPase 1	3.0E+06	7.0E+05	0.0169	3.5
P69905	HBA1	Hemoglobin subunit alpha	8.0E+08	4.0E+09	0.0179	0.2
P61803	DAD1	Dolichyl‐diphosphooligosaccharide–protein glycosyltransferase subunit DAD1	4.0E+06	1.0E+06	0.019	3.8
Q15063	POSTN	Periostin	1.0E+08	4.0E+07	0.0201	3.5
P01834	IGKC	Ig kappa chain C region	7.0E+07	2.0E+08	0.0202	0.4
P17174	GOT1	Aspartate aminotransferase, cytoplasmic	2.0E+07	7.0E+06	0.0223	2.4
P00915	CA1	Carbonic anhydrase 1	7.0E+06	4.0E+07	0.0232	0.2
P00450	CP	Ceruloplasmin	2.0E+07	5.0E+07	0.024	0.4
P02790	HPX	Hemopexin	5.0E+07	9.0E+07	0.0266	0.5
P17096	HMGA1	High mobility group protein HMG‐I/HMG‐Y	3.0E+07	6.0E+06	0.0271	5.2
Q15631	TSN	Translin	3.0E+06	7.0E+06	0.0287	0.5
P48163	ME1	NADP‐dependent malic enzyme	2.0E+06	5.0E+05	0.0289	3.6
Q16799	RTN1	Reticulon‐1	1.0E+07	5.0E+06	0.0307	2.5
P17677	GAP43	Neuromodulin	2.0E+08	9.0E+07	0.0311	2.1
O75348	ATP6V1G1	V‐type proton ATPase subunit G 1	9.0E+06	4.0E+06	0.034	2.1
O00154	ACOT7	Cytosolic acyl coenzyme A thioester hydrolase	1.0E+07	4.0E+06	0.0342	2.8
Q9NZN4	EHD2	EH domain‐containing protein 2	7.0E+06	5.0E+07	0.0347	0.1
P00747	PLG	Plasminogen	7.0E+06	2.0E+07	0.0357	0.4
P05156	CFI	Complement factor I	1.0E+06	7.0E+06	0.0368	0.2
O60716	CTNND1	Catenin delta‐1	3.0E+06	5.0E+05	0.0374	6.3
Q1KMD3	HNRNPUL2	Heterogeneous nuclear ribonucleoprotein U‐like protein 2	2.0E+06	4.0E+05	0.0377	4.2
P20591	MX1	Interferon‐induced GTP‐binding protein Mx1	8.0E+06	2.0E+06	0.0389	4.1
P40121	CAPG	Macrophage‐capping protein	1.0E+07	2.0E+06	0.0401	5.2
Q01118	SCN7A	Sodium channel protein type 7 subunit alpha	4.0E+06	2.0E+07	0.0408	0.2
Q86Y82	STX12	Syntaxin‐12	1.0E+06	0.0E+00	0.042	
Q9P0J7	KCMF1	E3 ubiquitin–protein ligase KCMF1	3.4E+05	0.0E+00	0.0426	
P05546	SERPIND1	Heparin cofactor 2	2.0E+06	7.0E+06	0.0493	0.3
P18509	ADCYAP1	Pituitary adenylate cyclase‐activating polypeptide	7.0E+06	0.0E+00	0.0499	

Proteins in bold were selected for IHC validation.

To identify enriched cellular functions and disease processes in invaded and noninvaded nerves, ingenuity pathway analysis (IPA) was performed. ‘Neurological Diseases’ was the most enriched disease category in addition to ‘Immunological Diseases’ and ‘Inflammatory Response’ (data not shown). Analysis of molecular and cellular functions revealed enrichment in ‘Cell Death and Survival’ (Fig. S2A), as well as ‘Cell Morphology’, ‘Cellular Growth and Proliferation’ and ‘Cell Movement’ functions, including several subcategories related to neuritogenesis and axonal development, comprising proteins such as VGF, Neuromodulin (GAP43), Laminin B1 and C1 (LAMB1 and LAMC1), Tenascin (TNC), serine–threonine protein kinase DCLK1, and microtubule‐associated protein 1B (MAP1B) (Fig. S2B).

### 3.2 Expression of SCG2 and VGF in the nerves

**Figure 2:**
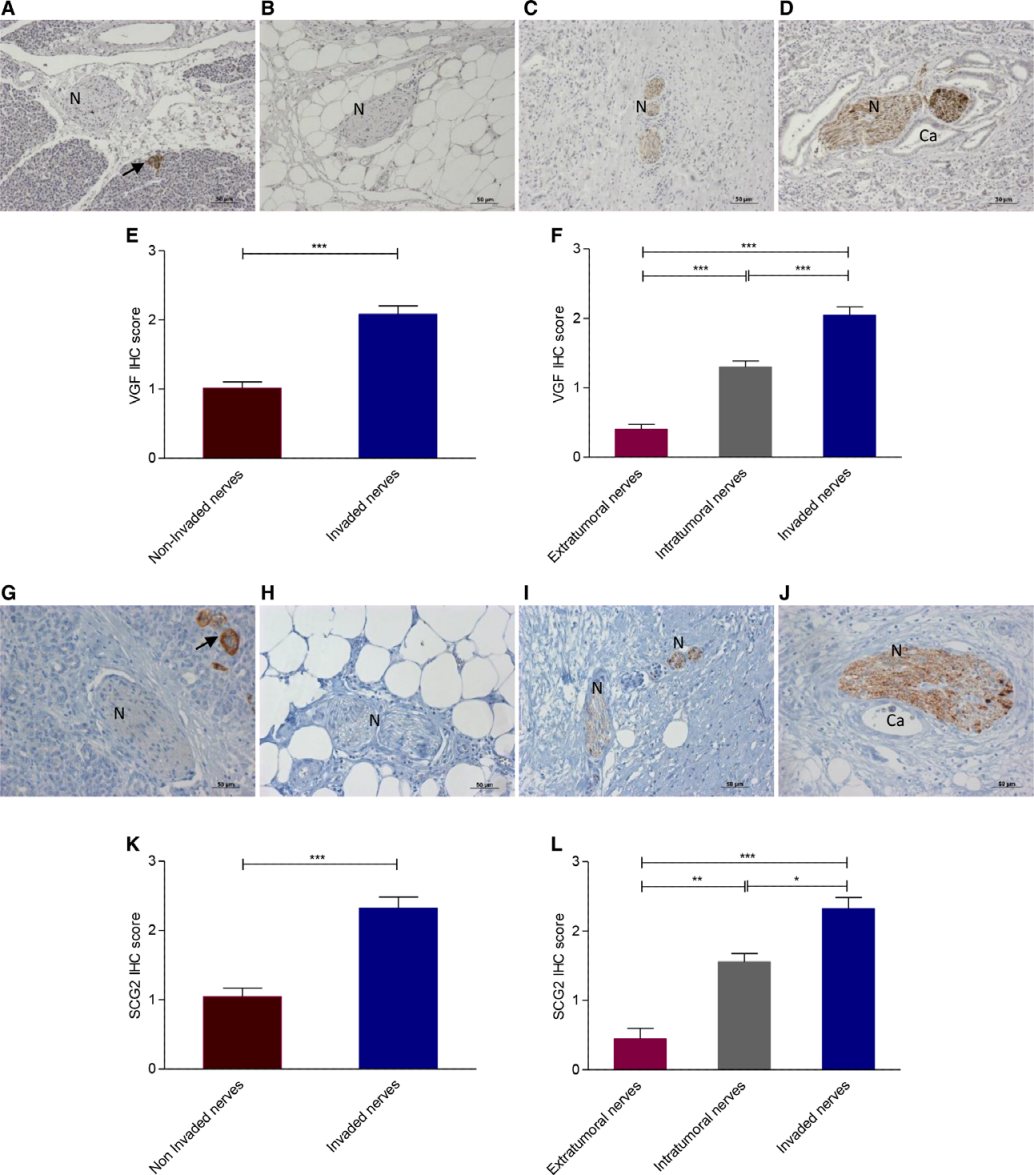
The VGF (A–F) and SCG2 (G–L) expression in pancreatic cancer. Both proteins are highly expressed in islets (arrow) (A/G). Representative images of PDAC nerves (N) illustrated a range of immunoreactivity. Extratumoral nerves typically showed no VGF (B) and SCG2 (H) expression, whereas intratumoral noninvaded nerves were weakly to moderately positive (C/I); invaded nerves were strongly immunoreactive (D/J). VGF and SCG2 and expression were significantly higher in invaded compared to noninvaded nerves (E/K) and also significantly different between the three groups of nerves in PDAC (F/L). Scale bar = 50 μm; 2E/K, Mann–Whitney test, ***P < 0.001, 2F/L, Kruskal–Wallis test, *P < 0.05, **P < 0.01, ***P < 0.001; error bars indicate SEM;* n* = 50.

Two of the highly up‐regulated proteins in the invaded compared to noninvaded nerves (Table 1), secretogranin II (SCG2) and neurosecretory protein VGF, were selected for further validation by immunohistochemistry. In normal pancreas, strong expression of both proteins was seen in the islets, while nerves were mostly devoid of any immunoreactivity (Fig. 2A/G). Nerves in PDAC tissues, however, showed a spectrum of SCG2 and VGF expression; the nerves outside the tumor/stromal mass did not usually express VGF or SCG2 (Fig. 2B/H), and nerves within the tumor showed weak to moderate expression (Fig. 2C/I), while nerves invaded with cancer displayed in the majority of cases moderate to strong expression (Fig. 2D/J) that was significantly higher than in noninvaded nerves (Fig. 2E/K). Based on this observed pattern, we classified nerves into noninvaded extratumoral and intratumoral nerves and invaded nerves; both SCG2 expression and VGF expression were significantly different between these three groups (Fig. 2F/L). These data are consistent with the proteomic results and suggest a role for PDAC cells in the induction of both proteins in pancreatic nerves.

### 3.3 *In vitro* modeling of neuronal plasticity in PNI

**Figure 3:**
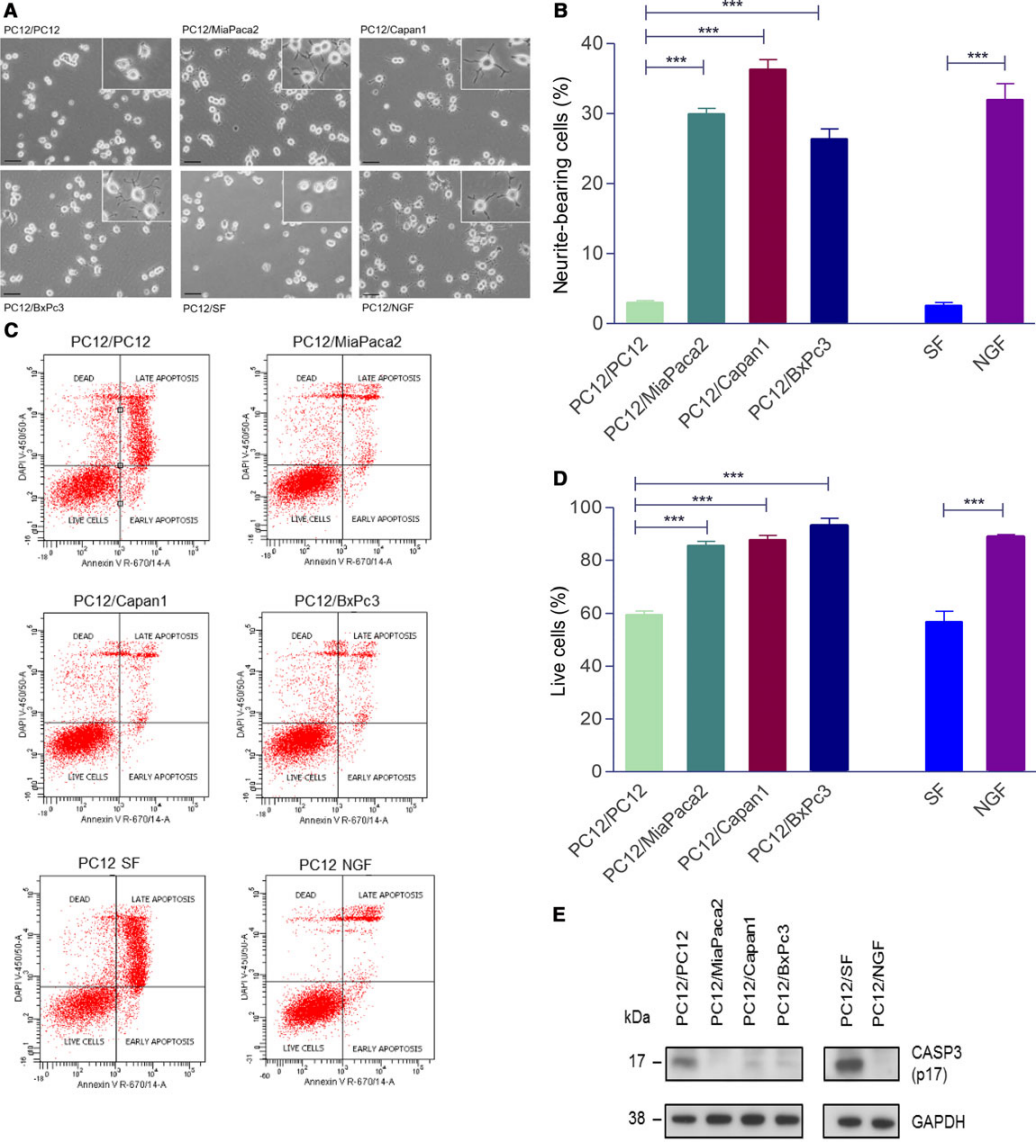
Neurite growth in Transwell co‐culture of PC12 cells with PDAC cell lines. PC12 co‐cultured on their own was used as controls. NGF (50 ng·mL^−1^) treatment was used as a positive control. Representative images of PC12 neurite growth in co‐culture with PDAC cell lines (A). Cells extending neurites longer than the maximum diameter of the cell body were counted and represented as a percentage of total number of cells (B). PC12 survival in transwell co‐cultures using Annexin V/DAPI flow cytometry assay. SF (serum‐free media) conditions were used as positive control for apoptosis, whereas CM (complete media) and NGF (50 ng·mL^−1^) were negative controls. Following 24 h co‐culture in serum‐free‐media, floating and attached PC12 cells were harvested, stained with Annexin and DAPI, and analyzed as detailed in Materials and methods section (C). The percentage of live cells (negative for both Annexin V and DAPI) in each condition was expressed relative to live cells in complete media (D). Western blot showing lower expression of Caspase 3 (E); scale bar = 50 μm; 3B/D, ANOVA, ***P < 0.001; error bars indicate SEM;* n* = 3.

In order to further validate molecular changes observed in proteomic analysis and facilitate characterization of underlying molecular mechanisms in PNI, we established an *in vitro* co‐culture model, using PC12 cells as the neuronal element. PC12 is a rat pheochromocytoma cell line which on exposure to NGF differentiates into sympathetic‐like neurons, exits the cell cycle, extends neurites, and becomes electrically excitable (Greene and Tischler, 1976). As the pancreas is innervated chiefly by the autonomic nervous system (sympathetic and parasympathetic) in addition to the intrinsic pancreatic ganglia (Kiba, 2004; Salvioli *et al*., 2002), PC12 cells represent an attractive model for these nerves. All three tested PDAC cell lines, MiaPaca2, Capan1, and BxPc3, induced neurite extension in PC12 cells in the Transwell co‐culture system, similar to that induced by NGF (Fig. 3A, B). The same cell lines also protected PC12 cells from serum starvation‐induced apoptosis, as measured by Annexin V/DAPI (Fig. 3C, D). In agreement with this, we observed an abrogation in the levels of cleaved Caspase 3 (CASP3) in PC12 cells when co‐cultured with MiaPaca2, Capan1, and BxPc3 using the Transwell co‐culture system, which was comparable to the effect of NGF (Fig. 3E). These results indicate that PDAC cells were able to induce neuronal plasticity in PC12 cells similar to that observed in PNI.

**Figure 4:**
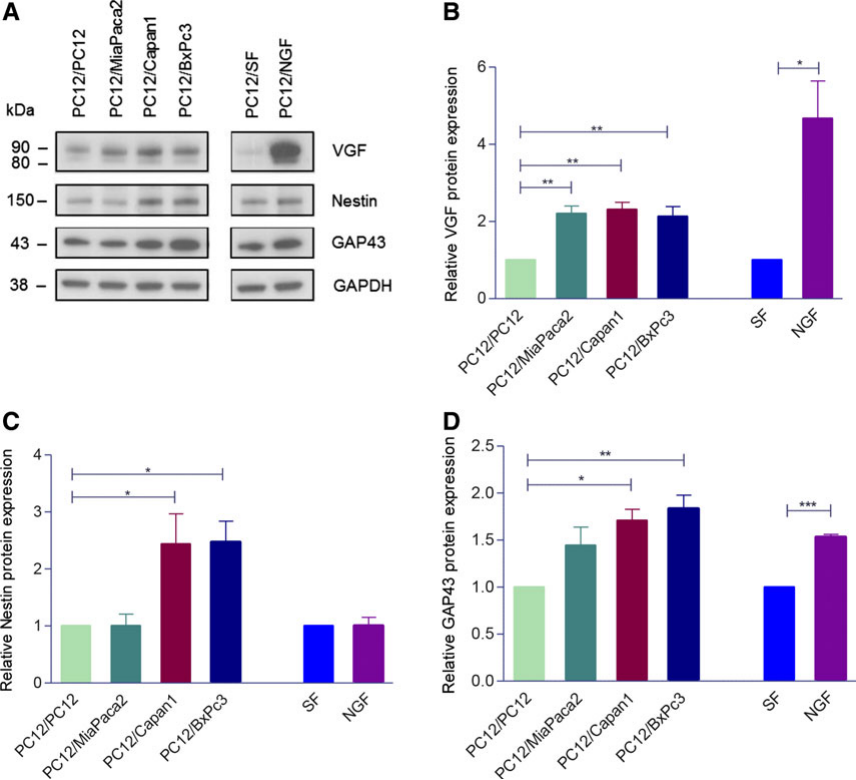
The VGF (A), Nestin, and GAP43 protein expression (C) in PC12 cells in Transwell co‐culture with PDAC cell lines or with PC12 as control. The representative western blots are shown. VGF appears as a doublet at ~80 and ~90 kDa, the lower band is thought to be a limited proteolytic product of the upper one (A). Densitometry analysis of three independent experiments (B, C, D). Bands are normalized to GAPDH as a loading control, and the results are expressed as fold change relative to control. 4B‐D, ANOVA, *P < 0.05; **P < 0.01; ***P < 0.001; error bars indicate SEM;* n* = 3.

Next, we assessed whether this model can also recapitulate the molecular changes seen in our proteomic analysis. In view of its role in neuronal plasticity (Alder *et al*., 2003), we tested VGF expression: Western blot showed twofold increase in VGF expression in PC12 cells co‐cultured with MiaPaca2, Capan1, and BxPC3 cells in the Transwell co‐cultures (Fig. 4A,B). Similar results were obtained at the mRNA level using qRT–PCR (Fig. S3). We also investigated whether PDAC cell lines are able to induce the expression of other up‐regulated proteins in invaded nerves seen in our proteomic analysis, namely Neuromodulin (GAP43) and Nestin (NES), as they have been previously linked with neuronal plasticity and their expression shown to be increased in nerves in PDAC using IHC (Ceyhan *et al*., 2009a; Kawamoto *et al*., 2009; Lenz *et al*., 2011). Indeed, the expression of both proteins significantly increased in PC12 cells co‐cultured with Capan1 or BxPc3 cells (Fig. 4A,C,D).

**Figure 5:**
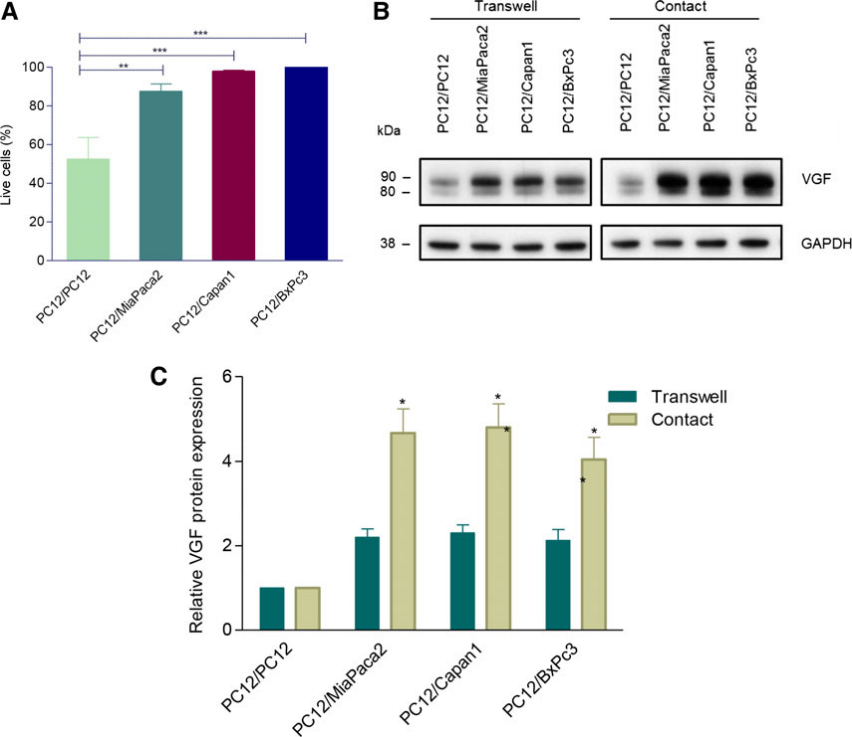
PC12 cell contact co‐cultures. PC12 cell survival measured using Annexin V/DAPI flow cytometry assay in contact co‐culture (A) and VGF protein expression in both transwell and contact co‐cultures (B, C). 5A/C, ANOVA, *P < 0.05; **P < 0.01; ***P < 0.001; error bars indicate SEM;* n* = 3.

Because our IHC analysis suggested a stronger induction of VGF immunoreactivity in the nerves as they come in close contact with cancer cells, we went on to verify whether *in vitro* contact co‐culture system could recapitulate this pattern of VGF expression. PC12 isolated from the contact co‐culture using FACS resulted in purity of the samples of 97% ±1.6 (mean ± SD) as estimated by FACS (Fig. S4), and >99% when estimated with anti‐CK19 antibody immunocytochemistry (Fig. S5). Similar to the observation in Transwell co‐cultures, PDAC cell lines in the contact co‐culture protected PC12 cells from serum starvation‐induced apoptosis (Fig. 5A). Interestingly, approximately twofold increase in the induction of VGF in PC12 cells was seen in contact compared to Transwell co‐cultures (Fig. 5B,C). This is therefore reminiscent of VGF expression pattern observed in pancreatic nerves in the context of PDAC.

**Figure 6:**
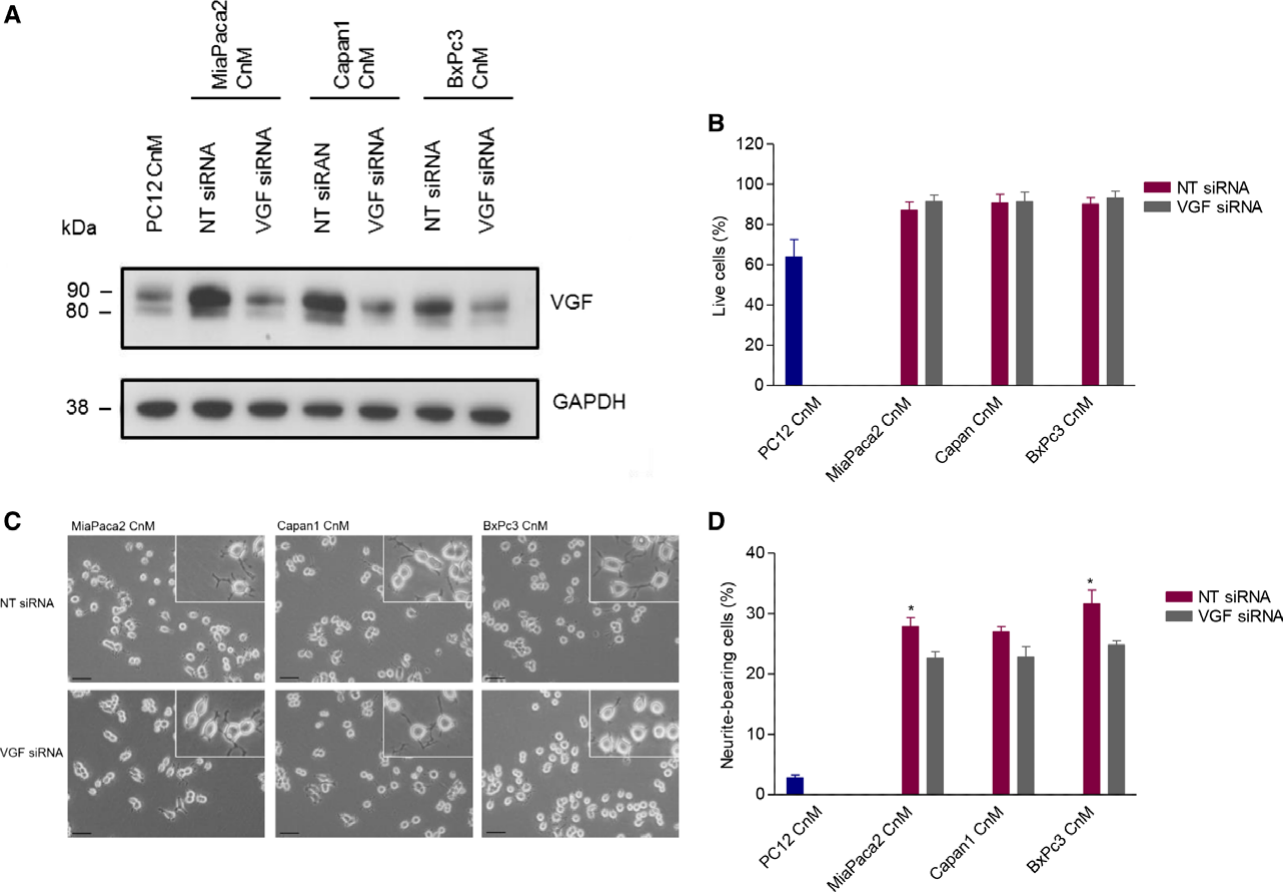
Prevention of VGF induction in PC12 cells using siRNA silencing. Western blot confirming the maintenance of baseline levels of VGF upon conditioned medium (CnM) following siRNA (A). PC12 cell survival (using Annexin V/DAPI flow cytometry) (B), representative pictures of PC12 cell neurite extension (C), and quantification of the neurite extension (D) following VGF siRNA or nontargeting siRNA treatment. Scale bar = 50μm; 6B/D, ANOVA, *P < 0.05; error bars indicate SEM;* n* = 3.

As VGF is known to play a role in cell survival (Severini *et al*., 2008) and neuronal plasticity (Alder *et al*., 2003), and its expression appears to mirror PC12 survival and neurite extension, we next tested whether VGF could mediate some of these phenotypic changes *in vitro*. While targeting VGF with specific siRNA successfully blocked its up‐regulation (Fig. 6A), this had no repercussion on survival of PC12 cells (Fig. 6B). However, PDAC cell‐induced neurite extension in PC12 cells was significantly attenuated after blocking VGF induction (Fig. 6C,D), indicating a functional role for VGF in the observed neuronal plasticity.

Taken together, data from our co‐culture system corroborate the proteomic profiling and IHC findings, confirm the ability of PDAC cells to induce neuronal plasticity, and reveal a functional role for VGF in this neuro‐epithelial interaction.

## 4 Discussion

Combining laser microdissection and proteomic approach has enabled us to perform the first direct characterization of the protein alterations in both cancer cells and nerves in the context of PNI in human PDAC. In the studies that investigated PNI and non‐PNI cancer cells using human tissues from cancers other than PDAC (Chen *et al*., 2007; Prueitt *et al*., 2008), a small number of differentially regulated genes were reported (none of these are in common with our proteomic data). While further in‐depth approach might be needed to reveal potentially subtler proteomic changes in cancer cells upon PNI in PDAC, these previous reports are confirmatory of largely similar profiles in PNI and non‐PNI cancer seen in our study.

In contrast, the analysis of the invaded and noninvaded nerves in PDAC revealed significant changes in their proteomic profiles, which *in silico* analysis highlighted to be a manifestation of neuronal plasticity. Interestingly, several up‐regulated proteins found here, including VGF and GAP43, pituitary adenylate cyclase‐activating polypeptide (ADCYAP1), Laminin A (LAMA), Nestin (NES), and TNC, have previously been shown to be up‐regulated in peripheral nerve injury (Costigan *et al*., 2002; Kim *et al*., 2009; Matsumura *et al*., 2010; Valder *et al*., 2003) and thus provide a molecular basis of the long‐described electron microscopy finding of nerve injury present within PNI (Bockman *et al*., 1994).

We identified several extracellular proteins as up‐regulated in invaded compared to noninvaded nerves (some of these were also seen in PNI compared to non‐PNI cancer), including laminins LAMA2, LAMB1, COL6A3, periostin (POST), nidogen 1 (NID1), and TNC. These are constitutive components of the extracellular milieu/matrix within which cancer cells and nerves are interacting, and some of them have dual roles in promoting both cancer growth and neurogenesis. This supports the concept that the PNI environment offers beneficial growth conditions for both cancer and nerves (Mancino *et al*., 2011).

Our proteomic analysis and IHC validation highlighted an increased expression of SCG2 and VGF in invaded compared to noninvaded nerves. Interestingly, the expression of both proteins increased as the nerves became closer to the cancer, which is consistent with previous observation of increasing neuroplasticity depending on the proximity of nerves to cancer (Ceyhan *et al*., 2010). SCG2 (secretogranin II, chromogranin C) is ~ 71‐kDa protein that belongs to the chromogranin–secretogranin family of acidic glycoproteins; it is expressed in secretory granules in a wide variety of peptide‐secreting cells throughout central nervous system, in scattered endocrine cells in stomach and duodenum and in pancreatic islets, as well as in neuronal elements throughout the intestinal wall (Boonen *et al*., 2007; Rosa *et al*., 1985). It is a principal target of REST (RE‐1 silencing transcription factor), an important regulator of neuronal differentiation which controls neurogenesis by preventing the differentiation of neural stem cells (Kim *et al*., 2015). SCG2 is a precursor for several peptides. One of them, EM66, is a 66‐amino acid anorexigenic neuropeptide that might be involved in regulation of feeding behavior and energy balance (Trebak *et al*., 2017); a 33‐amino acid neuropeptide called secretoneurin, on the other hand, promotes neurite outgrowth and repair of neuronal tissues (Shyu *et al*., 2008).

Similarly, VGF is expressed in both central and peripheral nervous system as well as in neuroendocrine cells in the gut (Levi *et al*., 2004; Lewis *et al*., 2015; Rindi *et al*., 2007; Salton *et al*., 2000). Its expression is regulated by neurotrophins during synaptic remodeling and axonal sprouting, and it is implicated in neuronal cell survival and plasticity (Alder *et al*., 2003; Severini *et al*., 2008; Shimazawa *et al*., 2010). Furthermore, akin to SCG2, VGF is also cleaved into several biologically active neuropeptides, expression of which varies between different tissues. Several VGF peptides were demonstrated to regulate nutrition and energy homeostasis, metabolism, and body weight (Bartolomucci *et al*., 2007; Watson *et al*., 2005). Furthermore, a central mechanistic role of VGF in neuropathic hypersensitivity and neuropathic pain has also recently been established (Ferri *et al*., 2011; Moss *et al*., 2008; Riedl *et al*., 2009), and it remains to be seen if VGF can be involved in generation of pain in PDAC patients as well.

Interestingly, this additional layer of functional complexity due to production of physiologically active peptides from both SCG2 and VGF is probably also the case in PDAC, which is corroborated in our proteomic dataset by the up‐regulation of PCSK1 in invaded nerves (Table 1), a known modulator of prohormone convertase 1 (PC1), which in turn is an enzyme responsible for both SCG2 and VGF processing.

In order to establish a simple and reproducible *in vitro* model of PNI, we have co‐cultured PC12 cells with PDAC cell lines. We show that tested PDAC cell lines were able to recapitulate the major phenotypic features of neuronal plasticity observed in tissues, namely nerve cell survival and neuritogenesis. PC12 cells undergo apoptosis in serum‐free media; NGF, bFGF and cAMP, known inducers of VGF have been shown to protect these cells from serum starvation‐mediated apoptosis (Rukenstein *et al*., 1991). We have therefore explored this further. While in our *in vitro* conditions VGF did not appear to mediate the survival of PC12 cells, blocking VGF induction resulted in significant reduction in neurite extension induced by both MiaPaca2‐ and BxPc3‐conditioned media. This indicated that while VGF is induced as part of the neuroplasticity response, being a secreted protein, it may also augment this process in an autocrine/paracrine fashion. While these experiments serve as a proof of principle and initial validation of usefulness of our *in vitro* model, further mechanistic studies detailing the roles of VGF (as well as SCG2) in PNI are now warranted.

Importantly, our model recapitulated several molecular changes observed in the proteomic analysis. PDAC cells were able to induce VGF expression mimicking the VGF induction in human tissues and also demonstrated their ability to up‐regulate VGF independently of any stromal influence. Interestingly, the up‐regulation of VGF in PC12 cells was even higher when they were in direct contact with cancer cells, which could be due to either exposure of PC12 cells to locally higher concentration of soluble paracrine factors or direct cell‐to‐cell communication. In addition to VGF, PDAC cell lines were also able to induce GAP43 as well as NES expression in PC12 cells, which was again consistent with our proteomic data. Both proteins have been shown previously by IHC to be expressed in nerves in PDAC (Ceyhan *et al*., 2009a; Kawamoto *et al*., 2009; Lenz *et al*., 2011). The induction of NES is particularly interesting, as it is a stem cell marker that is usually absent in adult postmitotic neuronal cells, indicating dedifferentiation response to nerve injury; its up‐regulation is also correlated with pain (Ceyhan *et al*., 2009b).

The functional and molecular alterations in PDAC cells in our co‐culture system still remain to be investigated. However, the flexibility of this model with regard to introducing different cellular components, performing gene manipulation and ease of collecting sufficiently large quantities of conditioned media from both cancer and PC12 cells (or any additional stromal or immune cellular component) can undoubtedly facilitate such investigations and underscores the advantages of this model when compared to primary neuronal cells and neural tissue explants. It would now be interesting to further develop this culture as a 3D model.

## 5 Conclusions

Here, we report on the first proteomes of both epithelial and neural components of PNI in human tissues and show that nerves invaded by pancreatic cancer undergo dramatic changes, manifested in neuronal plasticity, consistent with nerve injury. Some of the former changes were further successfully recapitulated in the developed *in vitro* co‐culture model, indicating a complex and intricate neuro‐epithelial crosstalk instigated during perineural invasion in pancreatic cancer.

## Conflict of interest

The authors declare no conflict of interest.

## Author contributions

WA and TCJ involved in study concept and design. WA, RJ, LD, TPR, and PRC involved in acquisition of data. WA, RJ, PRC, and TCJ analyzed and interpreted the data. WA and TCJ drafted the manuscript. WA, LD, IED, GOC, and TCJ critically revised the manuscript for important intellectual content. TCJ obtained funding. BD, IED, RMF, and GOC gave material support. RJ, PRC, and TCJ supervised the study.

## Supporting information


**Fig. S1**. Samples collected by laser microdissection. Matched PNI cancer cells, non‐PNI cancer cells, invaded nerves and non‐invaded nerves were laser microdissected from each of the five PDAC FFPE tissues. Top panel: before dissection; lower panel: verification of the dissected material.

**Fig. S2**. Ingenuity Pathway Analysis of differentially regulated proteins in the nerve samples showing neuritogenesis‐related functions and the proteins contributing to those functions (A) and several proteins contributing to the activation of cell survival and inhibition of apoptosis (B). Colours of the nodes reflect the level of activation/inhibition according to the colour key. The first number below the protein name indicates the p‐value and the second a fold change. The four arrows that connect the nodes illustrate the Predicted relationships in the functional networks: orange/blue, when leading to activation/inhibition of the downstream node (e.g.protein); yellow indicates that the found relationship in the submitted data are inconsistent with the prediction based on Ingenuity Knowledge Base; grey color indicates that no predictions could be made from our proteomics data based on Ingenuity Knowledge Base.

**Fig. S3**. VGF gene expression in PC12 cells co‐cultured with indicated PDAC cell lines. Values are relative to PC12 only co‐culture. ANOVA, *p<0.05, **p<0.01; error bars indicate SEM; n=3.

**Fig. S4**. . Representative example of FACS sorting of contact co‐cultures to isolate PC12 cells (P4, CMFDA stained) **(A).** Sorted PC12 cells were checked again using FACS to verify their purity **(B).**

**Fig. S5**. Immunocytochemistry staining of post‐sorted PC12 cells using anti‐CK19 antibody. Representative images (×400 magnification) of post‐sorted PC12 cells (right panel), the lack of CK19 staining indicates high degree of purity. PC12 cells and PDAC cells on their own were used as negative and positive controls, respectively (left panel). DAPI was used to stain nuclei.

**Table S1**. Clinical details of PDAC cases used for laser microdissection (highlighted in light green) and proteomics analysis. TNM: tumor size, lymph node, distant metastases. UICC: Union for International Cancer Control.

**Table S2**. List of 1,628 non‐redundant proteins identified in cancer invading the nerves (PNI 1‐5) and non‐invading cancer (non‐PNI 1‐5).

**Table S3**. List of differentially expressed proteins in invasive and non‐invasive cancer. PNI: invasive cancer in the nerves, non‐PNI cancer not invading nerves. FC: fold change. Two‐tailed student's t‐test.

**Table S4**. List of 923 non‐redundant proteins identified in invaded (IN 1‐5) and non‐invaded (NIN 1‐5) nerves.

**Table S5**. List of proteins up‐regulated in invaded nerves compared to non‐invaded nerves. FC: fold change. Two‐tailed student's t‐test.

## Data Availability

All data are provided with this submission and will be available freely after the acceptance of the manuscript.

## References

[mol212463-bib-0001] Abiatari I , DeOliveira T , Kerkadze V , Schwager C , Esposito I , Giese NA , Huber P , Bergman F , Abdollahi A , Friess H , Kleeff J (2009) Consensus transcriptome signature of perineural invasion in pancreatic carcinoma. Mol Cancer Ther 8, 1494–1504. 19509238 10.1158/1535-7163.MCT-08-0755

[mol212463-bib-0002] Alder J , Thakker‐Varia S , Bangasser DA , Kuroiwa M , Plummer MR , Shors TJ , Black IB (2003) Brain‐derived neurotrophic factor‐induced gene expression reveals novel actions of VGF in hippocampal synaptic plasticity. J Neurosci 23(34), 10800–10808. 14645472 10.1523/JNEUROSCI.23-34-10800.2003 PMC3374594

[mol212463-bib-0003] Amit M , Na'ara S , Gil Z (2016) Mechanisms of cancer dissemination along nerves. Nat Rev Cancer 16, 399–408. 27150016 10.1038/nrc.2016.38

[mol212463-bib-0004] Bapat AA , Hostetter G , Von Hoff DD and Han H (2011) Perineural invasion and associated pain in pancreatic cancer. Nat Rev Cancer 11(10), 695–707. 21941281 10.1038/nrc3131

[mol212463-bib-0005] Bartolomucci APR , Levi A , Pavone F and Moles A (2007) The role of the vgf gene and VGF‐derived peptides in nutrition and metabolism. Genes Nutr 2(2), 169–180. 18850173 10.1007/s12263-007-0047-0 PMC2474945

[mol212463-bib-0006] Bockman DE , Buchler M and Beger HG (1994) Interaction of pancreatic ductal carcinoma with nerves leads to nerve damage. Gastroenterology 107(1), 219–230. 8020665 10.1016/0016-5085(94)90080-9

[mol212463-bib-0007] Boonen K , Baggerman G , D'Hertog W , Husson SJ , Overbergh L , Mathieu C *et al* (2007) Neuropeptides of the islets of Langerhans: a peptidomics study. Gen Comp Endocrinol 152(2–3), 231–241. 17559849 10.1016/j.ygcen.2007.05.002

[mol212463-bib-0008] Ceyhan GO , Bergmann F , Kadihasanoglu M , Altintas B , Demir IE , Hinz U , Müller MW , Giese T , Büchler MW , Giese NA *et al* (2009a) Pancreatic neuropathy and neuropathic pain – a comprehensive pathomorphological study of 546 cases. Gastroenterology 136, 177–186 e1. 18992743 10.1053/j.gastro.2008.09.029

[mol212463-bib-0009] Ceyhan GO , Demir IE , Rauch U , Bergmann F , Muller MW , Buchler MW , Friess H , Schäfer KH (2009b) Pancreatic neuropathy results in “neural remodeling” and altered pancreatic innervation in chronic pancreatitis and pancreatic cancer. Am J Gast 104, 2555–2565. 10.1038/ajg.2009.380 19568227

[mol212463-bib-0010] Ceyhan GO , Schafer KH , Kerscher AG , Rauch U , Demir IE , Kadihasanoglu M , Böhm C , Müller MW , Büchler MW , Giese NA *et al* (2010) Nerve growth factor and artemin are paracrine mediators of pancreatic neuropathy in pancreatic adenocarcinoma. Ann Surg 251, 923–931. 20395845 10.1097/SLA.0b013e3181d974d4

[mol212463-bib-0011] Chen W , Zhang HL , Shao XJ , Jiang YG , Zhao XG , Gao X , Li JH , Yang J , Zhang YF , Liu BL *et al* (2007) Gene expression profile of salivary adenoid cystic carcinoma associated with perineural invasion. Tohoku J Exp Med 212, 319–334. 17592219 10.1620/tjem.212.319

[mol212463-bib-0012] Chou HH , Kuo CJ , Hsu JT , Chen TH , Lin CJ , Tseng JH , Yeh TS , Hwang TL , Jan YY (2013) Clinicopathologic study of node‐negative advanced gastric cancer and analysis of factors predicting its recurrence and prognosis. Am J Surg 205, 623–630. 23036602 10.1016/j.amjsurg.2012.04.014

[mol212463-bib-0013] Costigan M , Befort K , Karchewski L , Griffin RS , D'Urso D , Allchorne A , Sitarski J , Mannion JW , Pratt RE , Woolf CJ (2002) Replicate high‐density rat genome oligonucleotide microarrays reveal hundreds of regulated genes in the dorsal root ganglion after peripheral nerve injury. BMC Neurosci 3, 16. 12401135 10.1186/1471-2202-3-16 PMC139981

[mol212463-bib-0014] Cox J , Mann M (2008) MaxQuant enables high peptide identification rates, individualized p.p.b.‐range mass accuracies and proteome‐wide protein quantification. Nat Biotechnol 26, 1367–1372. 19029910 10.1038/nbt.1511

[mol212463-bib-0015] Dai H , Li R , Wheeler T , Ozen M , Ittmann M , Anderson M , Wang Y , Rowley D , Younes M , Ayala GE , (2007) Enhanced survival in perineural invasion of pancreatic cancer: an in vitro approach. Hum Pathol 38(2), 299–307. 17097719 10.1016/j.humpath.2006.08.002

[mol212463-bib-0016] Deborde S , Yu Y , Marcadis A , Chen CH , Fan N , Bakst RL , Wong RJ (2018) An in vivo sciatic nerve model of perineural invasion. J Vis Exp 134, e56857. 10.3791/56857 PMC5965264 29733315

[mol212463-bib-0017] Demir IE , Ceyhan GO , Liebl F , D'Haese JG , Maak M and Friess H (2012a) Neural invasion in pancreatic cancer: the past, present and future. Cancers 2(3), 1513–1527. 10.3390/cancers2031513 PMC3837319 24281170

[mol212463-bib-0018] Demir IE , Friess H and Ceyhan GO (2012b) Nerve‐cancer interactions in the stromal biology of pancreatic cancer. Front Physiol 3, 97. 22529816 10.3389/fphys.2012.00097 PMC3327893

[mol212463-bib-0019] Demir IE , Friess H and Ceyhan GO (2015) Neural plasticity in pancreatitis and pancreatic cancer. Nat Rev Gastroenterol Hepatol 12(11), 649–659. 26460352 10.1038/nrgastro.2015.166

[mol212463-bib-0020] D'Haese JG , Hartel M , Demir IE , Hinz U , Bergmann F , Buchler MW , Friess H , Ceyhan GO (2014) Pain sensation in pancreatic diseases is not uniform: the different facets of pancreatic pain. World J Gastroenterol 20(27), 9154–9161. 25083089 10.3748/wjg.v20.i27.9154 PMC4112857

[mol212463-bib-0021] Ferri G‐L , Noli B , Brancia C , D'Amato F and Cocco C (2011) VGF: An inducible gene product, precursor of a diverse array of neuro‐endocrine peptides and tissue‐specific disease biomarkers. J Chem Neuroanat 42(4), 249–261. 21621608 10.1016/j.jchemneu.2011.05.007

[mol212463-bib-0022] Greene LA and Tischler AS (1976) Establishment of a noradrenergic clonal line of rat adrenal pheochromocytoma cells which respond to nerve growth factor. Proc Natl Acad Sci USA 73(7), 2424–2428. 1065897 10.1073/pnas.73.7.2424 PMC430592

[mol212463-bib-0023] Hibi T , Mori T , Fukuma M , Yamazaki K , Hashiguchi A , Yamada T , Tanabe M , Aiura K , Kawakami T , Ogiwara A *et al* (2009) Synuclein‐gamma is closely involved in perineural invasion and distant metastasis in mouse models and is a novel prognostic factor in pancreatic cancer. Clin Cancer Res 15(8), 2864–2871. 19351749 10.1158/1078-0432.CCR-08-2946

[mol212463-bib-0024] Jobling P , Pundavela J , Oliveira SMR , Roselli S , Walker MM and Hondermarck H (2015) Nerve–cancer cell cross‐talk: a novel promoter of tumor progression. Can Res 75(9), 777–781. 10.1158/0008-5472.CAN-14-3180 25795709

[mol212463-bib-0025] Katoh H , Yasui H , Yamaguchi Y , Aoki J , Fujita H , Mori K , Negishi M (2000) Small GTPase RhoG is a key regulator for neurite outgrowth in PC12 cells. Mol Cell Biol 20(19), 7378–7387. 10982854 10.1128/mcb.20.19.7378-7387.2000 PMC86291

[mol212463-bib-0026] Kawamoto M , Ishiwata T , Cho K , Uchida E , Korc M , Naito Z , Tajiri T (2009) Nestin expression correlates with nerve and retroperitoneal tissue invasion in pancreatic cancer. Hum Pathol 40(2), 189–198. 18799194 10.1016/j.humpath.2008.02.022 PMC2654248

[mol212463-bib-0027] Kayahara M , Nagakawa T , Ueno K , Ohta T , Takeda T and Miyazaki I (1993) An evaluation of radical resection for pancreatic cancer based on the mode of recurrence as determined by autopsy and diagnostic imaging. Cancer 72(7), 2118–2123. 8104092 10.1002/1097-0142(19931001)72:7<2118::aid-cncr2820720710>3.0.co;2-4

[mol212463-bib-0028] Kelsen DP , Portenoy R , Thaler H , Tao Y and Brennan M (1997) Pain as a predictor of outcome in patients with operable pancreatic carcinoma. Surgery 122(1), 53–59. 9225915 10.1016/s0039-6060(97)90264-6

[mol212463-bib-0029] Kiba T (2004) Relationships between the autonomic nervous system and the pancreas including regulation of regeneration and apoptosis: recent developments. Pancreas 29(2), e51–e58. 15257115 10.1097/00006676-200408000-00019

[mol212463-bib-0030] Kim HJ , Denli AM , Wright R , Baul TD , Clemenson GD , Morcos AS , Zhao C , Schafer ST , Gage FH , Kagalwala MN (2015) REST regulates non‐cell‐autonomous neuronal differentiation and maturation of neural progenitor cells via secretogranin II. J Neurosci 35, 14872–14884. 26538656 10.1523/JNEUROSCI.4286-14.2015 PMC4635134

[mol212463-bib-0031] Kim DS , Figueroa KW , Li KW , Boroujerdi A , Yolo T and Luo ZD (2009) Profiling of dynamically changed gene expression in dorsal root ganglia post peripheral nerve injury and a critical role of injury‐induced glial fibrillary acidic protein in maintenance of pain behaviors [corrected]. Pain 143(1–2), 114–122. 19307059 10.1016/j.pain.2009.02.006 PMC2743568

[mol212463-bib-0032] Koide N , Yamada T , Shibata R , Mori T , Fukuma M , Yamazaki K , Aiura K , Shimazu M , Hirohashi S , Nimura Y *et al* (2006) Establishment of perineural invasion models and analysis of gene expression revealed an invariant chain (CD74) as a possible molecule involved in perineural invasion in pancreatic cancer. Clin Cancer Res 12(8), 2419–2426. 16638847 10.1158/1078-0432.CCR-05-1852

[mol212463-bib-0033] Lenz J , Karasek P , Jarkovsky J , Muckova K , Dite P , Kala Z , Veselska R , Hermanova M Z (2011) Clinicopathological correlations of nestin expression in surgically resectable pancreatic cancer including an analysis of perineural invasion. J Gast Liver Dis 20(4), 389–396. 22187705

[mol212463-bib-0034] Levi A , Ferri G‐L , Watson E , Possenti R and Salton SRJ (2004) Processing, distribution, and function of VGF, a neuronal and endocrine peptide precursor. Cell Mol Neurobiol 24(4), 517–533. 15233376 10.1023/B:CEMN.0000023627.79947.22 PMC11529936

[mol212463-bib-0035] Lewis JE , Brameld JM and Jethwa PH (2015) Neuroendocrine role for VGF. Front Endocrinol 6, 3. 10.3389/fendo.2015.00003 PMC4313783 25699015

[mol212463-bib-0036] Liebig C , Ayala G , Wilks JA , Berger DH and Albo D (2009) Perineural invasion in cancer: a review of the literature. Cancer 115, 3379–3391. 19484787 10.1002/cncr.24396

[mol212463-bib-0037] Liu BLK (2002) Neural invasion in pancreatic carcinoma. Hepatobiliary Pancreat Dis Int 1(3), 469–476. 14607730

[mol212463-bib-0038] Mancino M , Ametller E , Gascón P , Almendro V (2011) The neuronal influence on tumor progression. Biochim et Biophys Acta – Rev Cancer 1816, 105–118. 10.1016/j.bbcan.2011.04.005 21616127

[mol212463-bib-0039] Marchesi F , Piemonti L , Mantovani A and Allavena P (2010) Molecular mechanisms of perineural invasion, a forgotten pathway of dissemination and metastasis. Cytokine Growth Factor Rev 21, 77–82. 20060768 10.1016/j.cytogfr.2009.11.001

[mol212463-bib-0040] Matsumura S , Takagi K , Okuda‐Ashitaka E , Lu J , Naritsuka H , Yamaguchi M , Ito S (2010) Characterization of nestin expression in the spinal cord of GFP transgenic mice after peripheral nerve injury. Neuroscience 170(3), 942–953. 20673789 10.1016/j.neuroscience.2010.07.034

[mol212463-bib-0041] Moss A , Ingram R , Koch S , Theodorou A , Low L , Baccei M , Hathway GJ , Costigan M , Salton SR , Fitzgerald M (2008) Origins, actions and dynamic expression patterns of the neuropeptide VGF in rat peripheral and central sensory neurones following peripheral nerve injury. Mol Pain 4(1), 62. 19077191 10.1186/1744-8069-4-62 PMC2614976

[mol212463-bib-0042] Pour PM , Bell RH and Batra SK (2003) Neural invasion in the staging of pancreatic cancer. Pancreas 26, 322–325. 12717262 10.1097/00006676-200305000-00002

[mol212463-bib-0043] Prueitt RL , Yi M , Hudson RS , Wallace TA , Howe TM , Yfantis HG , Lee DH , Stephens RM , Liu CG , Calin GA *et al* (2008) Expression of microRNAs and protein‐coding genes associated with perineural invasion in prostate cancer. Prostate 68, 1152–1164. 18459106 10.1002/pros.20786 PMC2597330

[mol212463-bib-0044] Renz BW , Takahashi R , Tanaka T , Macchini M , Hayakawa Y , Dantes Z , Maurer HC , Chen X , Jiang Z , Westphalen CB *et al* (2018b) β2 Adrenergic‐neurotrophin feedforward loop promotes pancreatic cancer. Cancer Cell 33(1), 75–90.e7. 29249692 10.1016/j.ccell.2017.11.007 PMC5760435

[mol212463-bib-0045] Renz BW , Tanaka T , Sunagawa M , Takahashi R , Jiang Z , Macchini M , Dantes Z , Valenti G , White RA , Middelhoff MA *et al* (2018a) Cholinergic signaling via muscarinic receptors directly and indirectly suppresses pancreatic tumorigenesis and cancer stemness. Cancer Discov 8, 1458–1473. 30185628 10.1158/2159-8290.CD-18-0046 PMC6214763

[mol212463-bib-0046] Riedl MS , Braun PD , Kitto KF , Roiko SA , Anderson LB , Honda CN , Fairbanks CA , Vulchanova L (2009) Proteomic analysis uncovers novel actions of the neurosecretory protein VGF in nociceptive processing. J Neurosci 29(42), 13377–13388. 19846725 10.1523/JNEUROSCI.1127-09.2009 PMC2801058

[mol212463-bib-0047] Rindi G , Licini L , Necchi V , Bottarelli L , Campanini N , Azzoni C , Favret M , Giordano G , D'Amato F , Brancia C *et al* (2007) Peptide products of the neurotrophin‐inducible gene vgf are produced in human neuroendocrine cells from early development and increase in hyperplasia and neoplasia. J Clin Endocrinol Metab 92(7), 2811–2815. 17440014 10.1210/jc.2007-0035

[mol212463-bib-0048] Rosa P , Hille A , Lee RW , Zanini A , De Camilli P and Huttner WB (1985) Secretogranins I and II: two tyrosine‐sulfated secretory proteins common to a variety of cells secreting peptides by the regulated pathway. J Cell Biol 101, 1999–2011. 4055903 10.1083/jcb.101.5.1999 PMC2113975

[mol212463-bib-0049] Rukenstein A , Rydel RE and Greene LA (1991) Multiple agents rescue PC12 cells from serum‐free cell death by translation‐ and transcription‐independent mechanisms. J Neurosci 11(8), 2552–2563. 1714494 10.1523/JNEUROSCI.11-08-02552.1991 PMC6575525

[mol212463-bib-0050] Saloman JL , Albers KM , Li D , Hartman DJ , Crawford HC , Muha EA , Rhim AD , Davis BM (2016) Ablation of sensory neurons in genetic model of pancreatic ductal adenocarcinoma slows initiation and progression of cancer. PNAS 113(11), 3078–3083. 26929329 10.1073/pnas.1512603113 PMC4801275

[mol212463-bib-0051] Saloman JL , Singhi AD , Hartman DJ , Normolle DP , Albers KM and Davis BM (2018) Systemic depletion of nerve growth factor inhibits disease progression in a genetically engineered model of pancreatic ductal adenocarcinoma. Pancreas 47(7), 856–863. 29975347 10.1097/MPA.0000000000001090 PMC6044729

[mol212463-bib-0052] Salton SR , Ferri GL , Hahm S , Snyder SE , Wilson AJ , Possenti R , Levi A (2000) VGF: a novel role for this neuronal and neuroendocrine polypeptide in the regulation of energy balance. Front Neuroendocrinol 21(3), 199–219. 10882540 10.1006/frne.2000.0199

[mol212463-bib-0053] Salvioli B , Bovara M , Barbara G , De Ponti F , Stanghellini V , Tonini M , Guerrini S , Cremon C , Degli Esposti M , Koumandou M *et al* (2002) Neurology and neuropathology of the pancreatic innervation. JOP 3(2), 26–33. 11884764

[mol212463-bib-0054] Severini C , Ciotti MT , Biondini L , Quaresima S , Rinaldi AM , Levi A , Frank C , Possenti R (2008) TLQP‐21, a neuroendocrine VGF‐derived peptide, prevents cerebellar granule cells death induced by serum and potassium deprivation. J Neurochem 104(2), 534–544. 18173805 10.1111/j.1471-4159.2007.05068.x

[mol212463-bib-0055] Shimada K , Nara S , Esaki M , Sakamoto Y , Kosuge T and Hiraoka N (2011) Intrapancreatic nerve invasion as a predictor for recurrence after pancreaticoduodenectomy in patients with invasive ductal carcinoma of the pancreas. Pancreas 40(3), 464–468. 21289526 10.1097/MPA.0b013e31820b5d37

[mol212463-bib-0056] Shimazawa M , Tanaka H , Ito Y , Morimoto N , Tsuruma K , Kadokura M , Tamura S , Inoue T , Yamada M , Takahashi H *et al* (2010) An inducer of VGF protects cells against ER stress‐induced cell death and prolongs survival in the mutant SOD1 animal models of familial ALS. PLoS ONE 5(12), e15307. 21151573 10.1371/journal.pone.0015307 PMC3000345

[mol212463-bib-0057] Shyu WC , Lin SZ , Chiang MF , Chen DC , Su CY , Wang HJ , Liu RS , Tsai CH , Li H (2008) Secretoneurin promotes neuroprotection and neuronal plasticity via the Jak2/Stat3 pathway in murine models of stroke. J Clin Invest 118(1), 133–148. 18079966 10.1172/JCI32723 PMC2129236

[mol212463-bib-0058] Stopczynski RE , Normolle DP , Hartman DJ , Ying H , DeBerry JJ , Bielefeldt K , Rhim AD , DePinho RA , Albers KM , Davis BM (2014) Neuroplastic changes occur early in the development of pancreatic ductal adenocarcinoma. Cancer Res 74(6), 1718–1727. 24448244 10.1158/0008-5472.CAN-13-2050 PMC4036226

[mol212463-bib-0059] Trebak F , Dubuc I , Arabo A , Alaoui A , Boukhzar L , Maucotel J , Picot M , Cherifi S , Duparc C , Leprince J *et al* (2017) A potential role for the secretogranin II‐derived peptide EM66 in the hypothalamic regulation of feeding behaviour. J Neuroendocrinol 29, 1–11. 10.1111/jne.12459 28166374

[mol212463-bib-0060] Valder CR , Liu JJ , Song YH and Luo ZD (2003) Coupling gene chip analyses and rat genetic variances in identifying potential target genes that may contribute to neuropathic allodynia development. J Neurochem 87(3), 560–573. 14535940 10.1046/j.1471-4159.2003.02016.x

[mol212463-bib-0061] Watson E , Hahm S , Mizuno TM , Windsor J , Montgomery C , Scherer PE , Mobbs CV , Salton SR (2005) VGF ablation blocks the development of hyperinsulinemia and hyperglycemia in several mouse models of obesity. Endocrinology 146(12), 5151–5163. 16141392 10.1210/en.2005-0588

[mol212463-bib-0062] Whiteman HJ , Weeks ME , Dowen SE , Barry S , Timms JF , Lemoine NR , Crnogorac‐Jurcevic T (2007) The role of S100P in the invasion of pancreatic cancer cells is mediated through cytoskeletal changes and regulation of cathepsin D. Cancer Res 67(18), 8633–8642. 17875703 10.1158/0008-5472.CAN-07-0545

[mol212463-bib-0063] Zhang JF , Hua R , Sun YW , Liu W , Huo YM , Liu DJ , Li J (2013) Influence of perineural invasion on survival and recurrence in patients with resected pancreatic cancer. Asian Pacific J Cancer Prevent 14(9), 5133–5139. 10.7314/apjcp.2013.14.9.5133 24175789

