# Supplementary figures and images for: Perineural invasion in pancreatic cancer: proteomic analysis and in vitro modelling
Source: Mol Oncol. 2019 Mar 5;13(5):1075–91. doi: 10.1002/1878-0261.12463 (PMC6487729; doi:10.1002/1878-0261.12463)

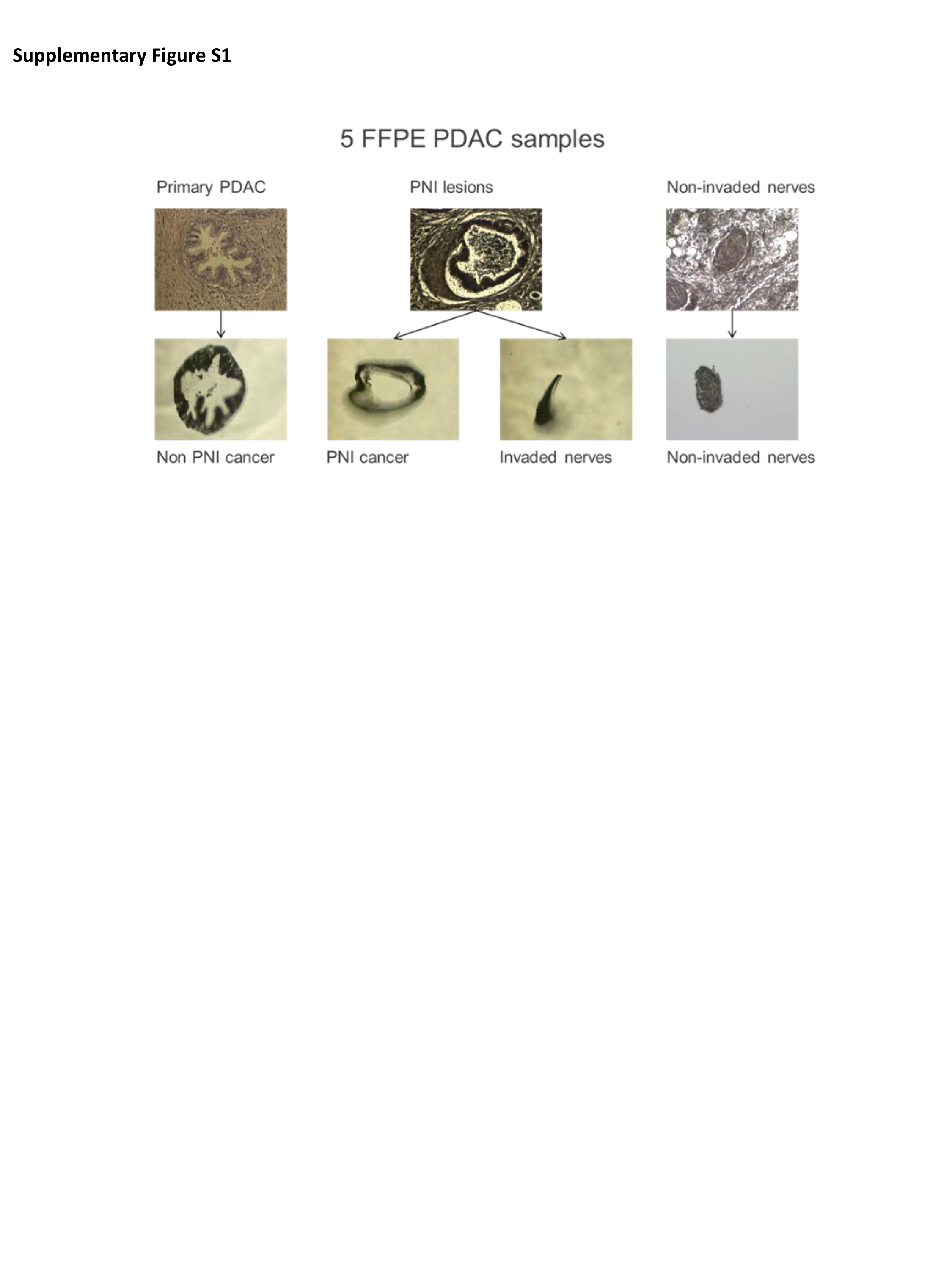

Supplement: Supplementary file 1 — Fig. S1. Samples collected by laser microdissection. Matched PNI cancer cells, non‐PNI cancer cells, invaded nerves and non‐invaded nerves were laser microdissected from each of the five PDAC FFPE tissues. Top panel: before dissection; lower panel: verification of the dissected material. [file MOL2-13-1075-s001.tif]

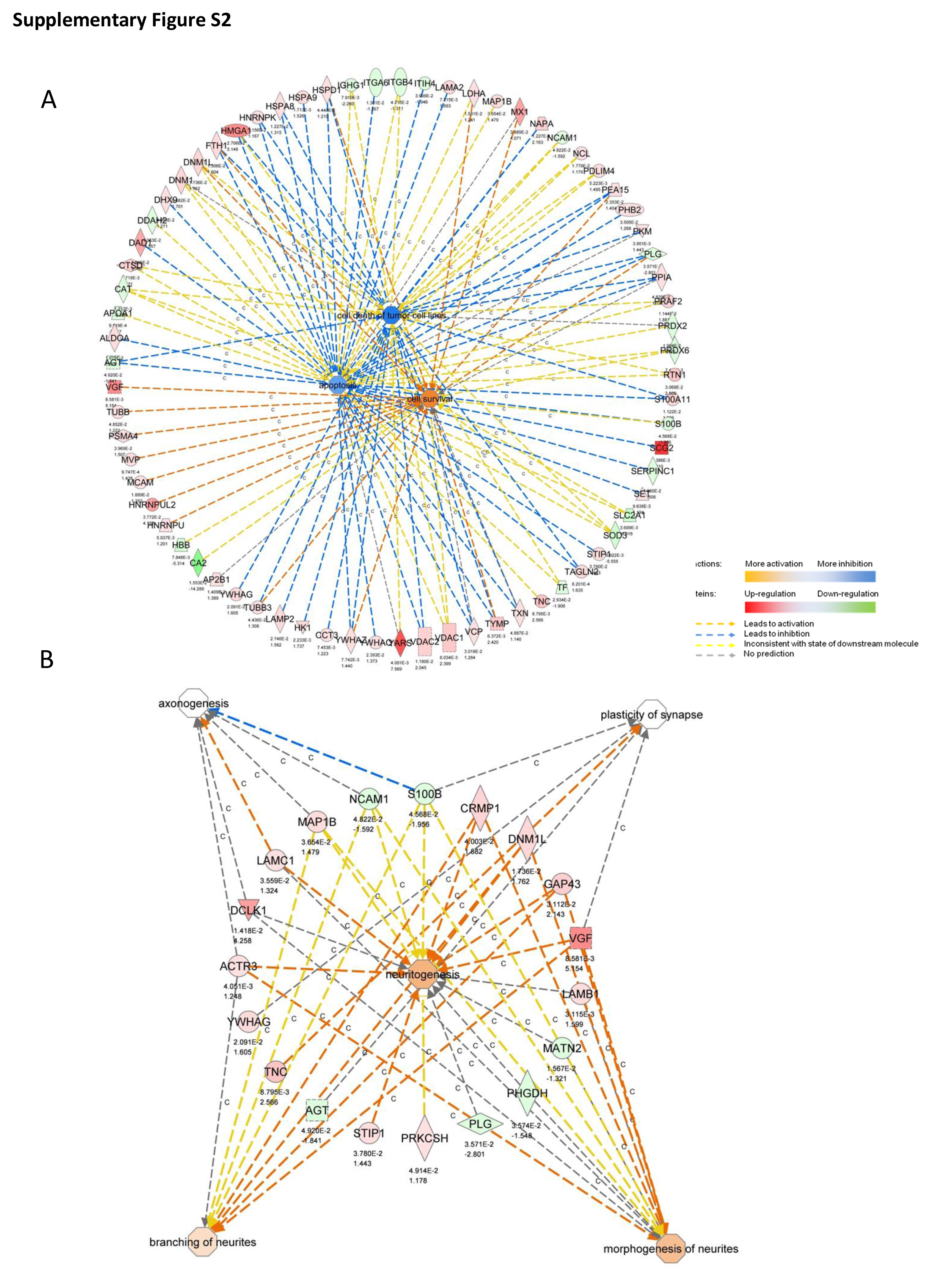

Supplement: Supplementary file 2 — Fig. S2. Ingenuity Pathway Analysis of differentially regulated proteins in the nerve samples showing neuritogenesis‐related functions and the proteins contributing to those functions (A) and several proteins contributing to the activation of cell survival and inhibition of apoptosis (B). Colours of the nodes reflect the level of activation/inhibition according to the colour key. The first number below the protein name indicates the p‐value and the second a fold change. The four arrows that connect the nodes illustrate the Predicted relationships in the functional networks: orange/blue, when leading to activation/inhibition of the downstream node (e.g.protein); yellow indicates that the found relationship in the submitted data are inconsistent with the prediction based on Ingenuity Knowledge Base; grey color indicates that no predictions could be made from our proteomics data based on Ingenuity Knowledge Base. [file MOL2-13-1075-s002.tif]

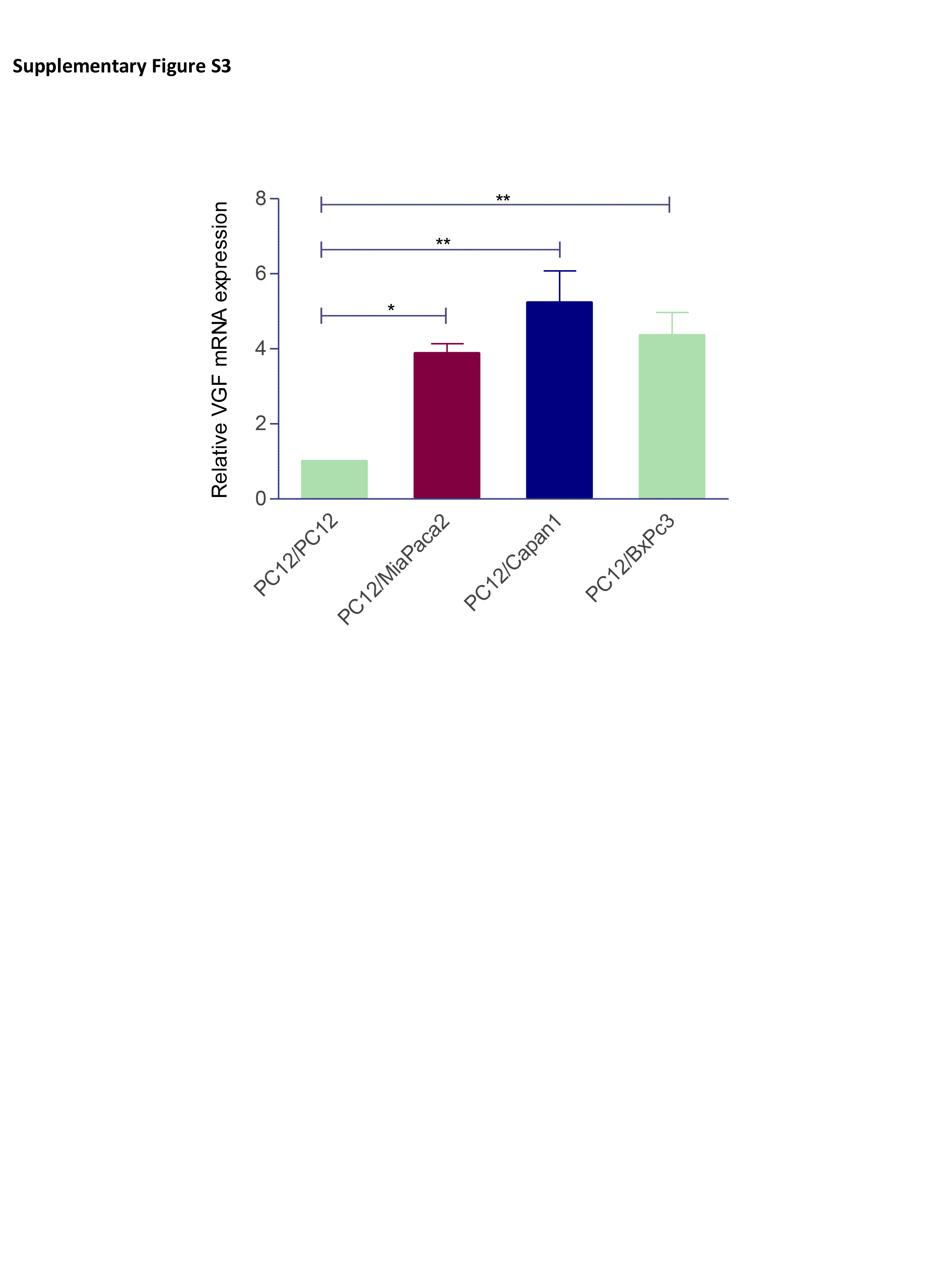

Supplement: Supplementary file 3 — Fig. S3. VGF gene expression in PC12 cells co‐cultured with indicated PDAC cell lines. Values are relative to PC12 only co‐culture. ANOVA, *p<0.05, **p<0.01; error bars indicate SEM; n=3. [file MOL2-13-1075-s003.tif]

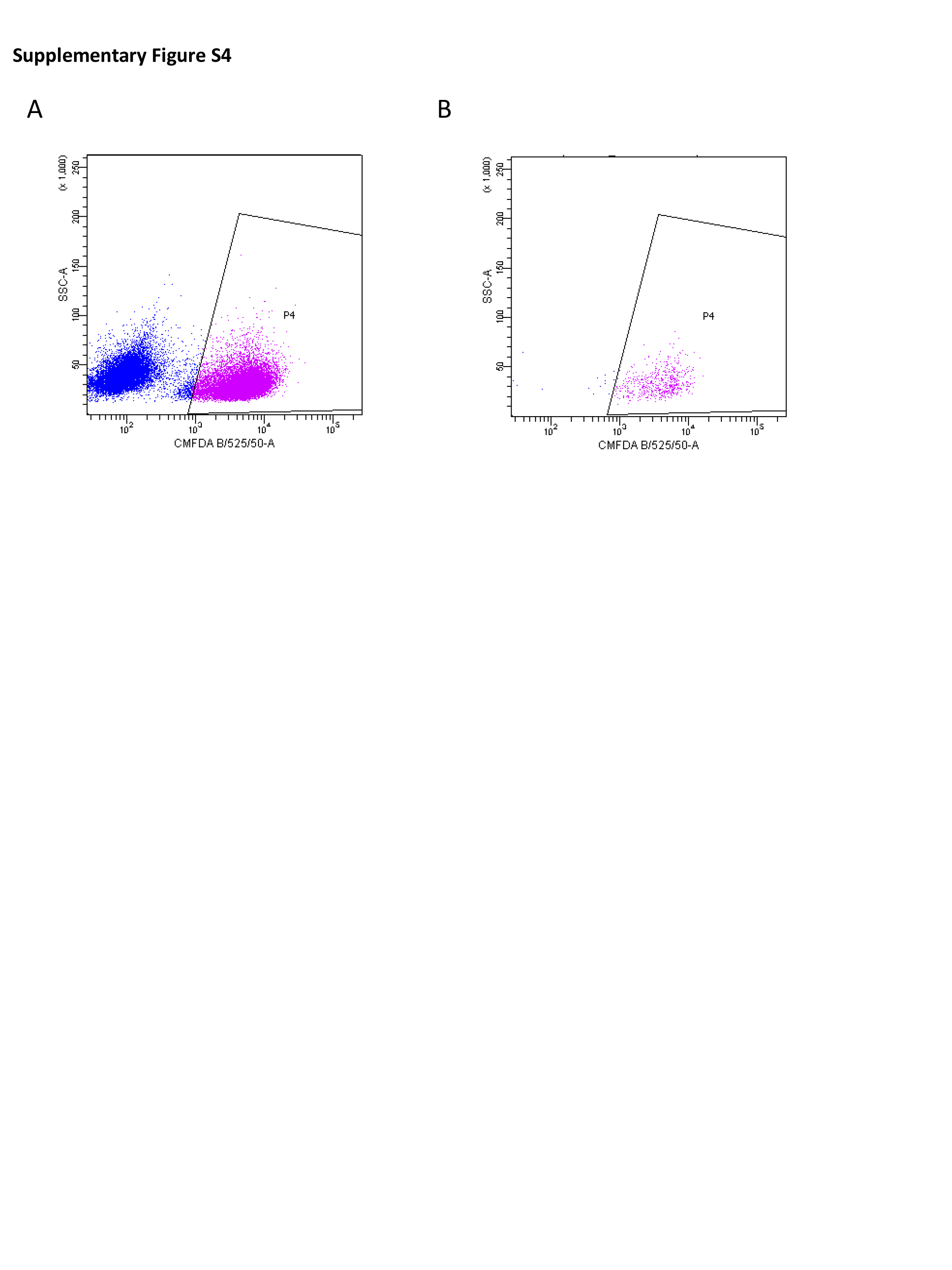

Supplement: Supplementary file 4 — Fig. S4. . Representative example of FACS sorting of contact co‐cultures to isolate PC12 cells (P4, CMFDA stained) (A). Sorted PC12 cells were checked again using FACS to verify their purity (B). [file MOL2-13-1075-s004.tif]

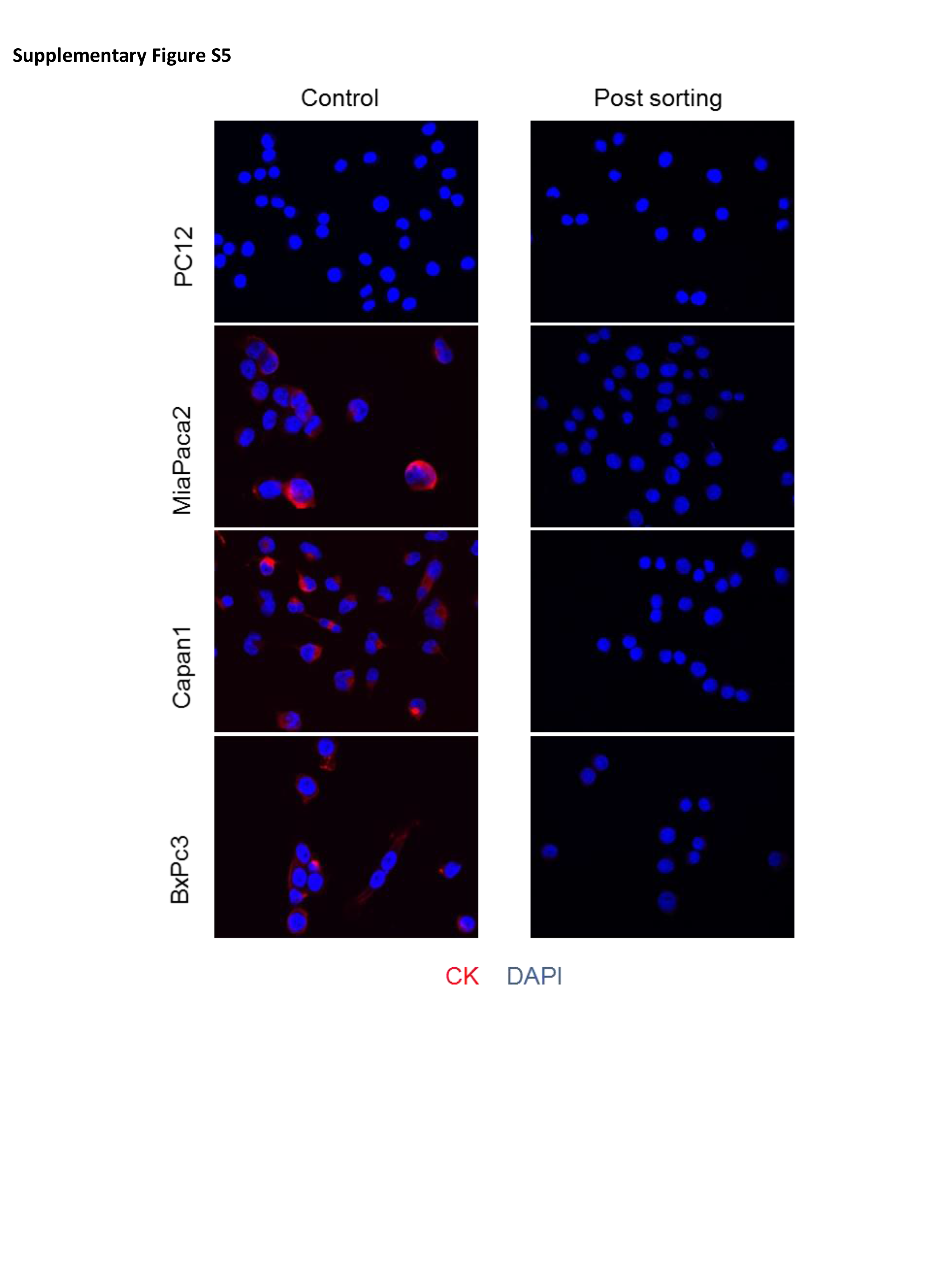

Supplement: Supplementary file 5 — Fig. S5. Immunocytochemistry staining of post‐sorted PC12 cells using anti‐CK19 antibody. Representative images (×400 magnification) of post‐sorted PC12 cells (right panel), the lack of CK19 staining indicates high degree of purity. PC12 cells and PDAC cells on their own were used as negative and positive controls, respectively (left panel). DAPI was used to stain nuclei. [file MOL2-13-1075-s005.tif]
